# Supplementary material for: Intramolecular autoinhibition regulates the selectivity of PRPF40A tandem WW domains for proline-rich motifs
Source: Nat Commun. 2024 May 8;15:3888. doi: 10.1038/s41467-024-48004-x (PMC11079029; doi:10.1038/s41467-024-48004-x)
Supplement: Supplementary file 1 — Supplementary Information [file 41467_2024_48004_MOESM1_ESM.pdf]

Supplementary Information

Intramolecular autoinhibition regulates the selectivity of PRPF40A tandem WW domains for proline-rich motifs

Santiago Martínez-Lumbreras[1,2,*], Lena K. Träger[2], Miriam M. Mulorz[3], Marco Payr[2], Varvara Dikaya[2], Clara Hipp[1,2], Julian König[3] and Michael Sattler[1,2,*]

Table to contents

Supplementary Figures

Supplementary Figure 1 3
Supplementary Figure 2 4
Supplementary Figure 3 6
Supplementary Figure 4 7
Supplementary Figure 5 8
Supplementary Figure 6 9
Supplementary Figure 7 11
Supplementary Figure 8 12
Supplementary Figure 9 13
Supplementary Figure 10 14
Supplementary Figure 11 15
Supplementary Figure 12 16
Supplementary Figure 13 17
Supplementary Figure 14 18
Supplementary Figure 15 19
Supplementary Figure 16 20
Supplementary Figure 17 21
Supplementary Figure 18 22
Supplementary Figure 19 23
Supplementary Figure 20 24
Supplementary Figure 21 25
Supplementary Figure 22 26
Supplementary Figure 23 28
Supplementary Figure 24 29
Supplementary Figure 25 30
Supplementary Figure 26 31
Supplementary Figure 27 32
Supplementary Figure 28 33
Supplementary Figure 29 34

Supplementary Tables

Supplementary Table 1 35
Supplementary Table 2 36
Supplementary Table 3 37
Supplementary Table 4 38
Supplementary Table 5 39
Supplementary Table 6 40

SUPPLEMENTARY FIGURES

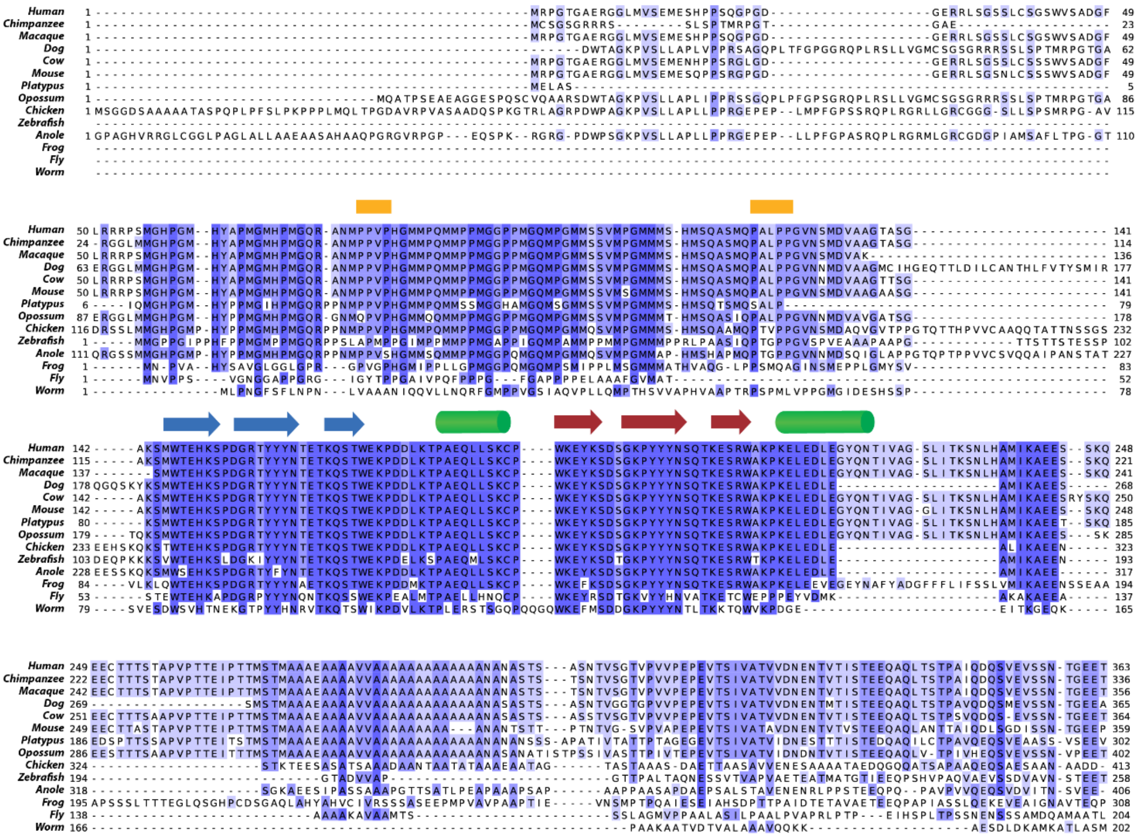

Supplementary Figure 1. Sequence alignment of the N-terminal region of PRPF40A for several animal species: *Homo sapiens* (Human), *Pan troglodytes* (Chimpanzee), *Macaca mulatta* (Macaque), *Canis lupus familiaris* (Dog), *Bos taurus* (Cow), *Mus musculus* (Mouse), *Ornithorhynchus anatius* (Platypus), *Monodelphis domestica* (Opossum), *Gallus gallus* (Chicken), *Danio rerio* (Zebrafish), *Anolis carolinensis* (Anole), *Xenoupus tropicalis* (Frog), *Drosophila melanogaster* (Fly) and *Caenorhabditis elegans* (Worm). Protein database codes are as follow: Human (O75400), Chimpanzee (H2R0E8), Macaque (A0A5F7ZXB1), Dog (J9P4S4), Cow (G3MXT3), Mouse (Q9R1C7), Platypus (F6WU22), Opossum (F6QNU6), Chicken (F1NCE3), Zebrafish (Q7ZUE4), Anole (H9GJG6), Frog (F7ESA8), Fly (Q9VQK5) and Worm (P34600). The secondary structure elements of the WW domain tandem have been depicted (blue and red – β-strands of WW1 and WW2, green – linker and C-terminal α-helices); the two autoinhibitory proline rich regions in higher eukaryotes are marked with yellow bars.

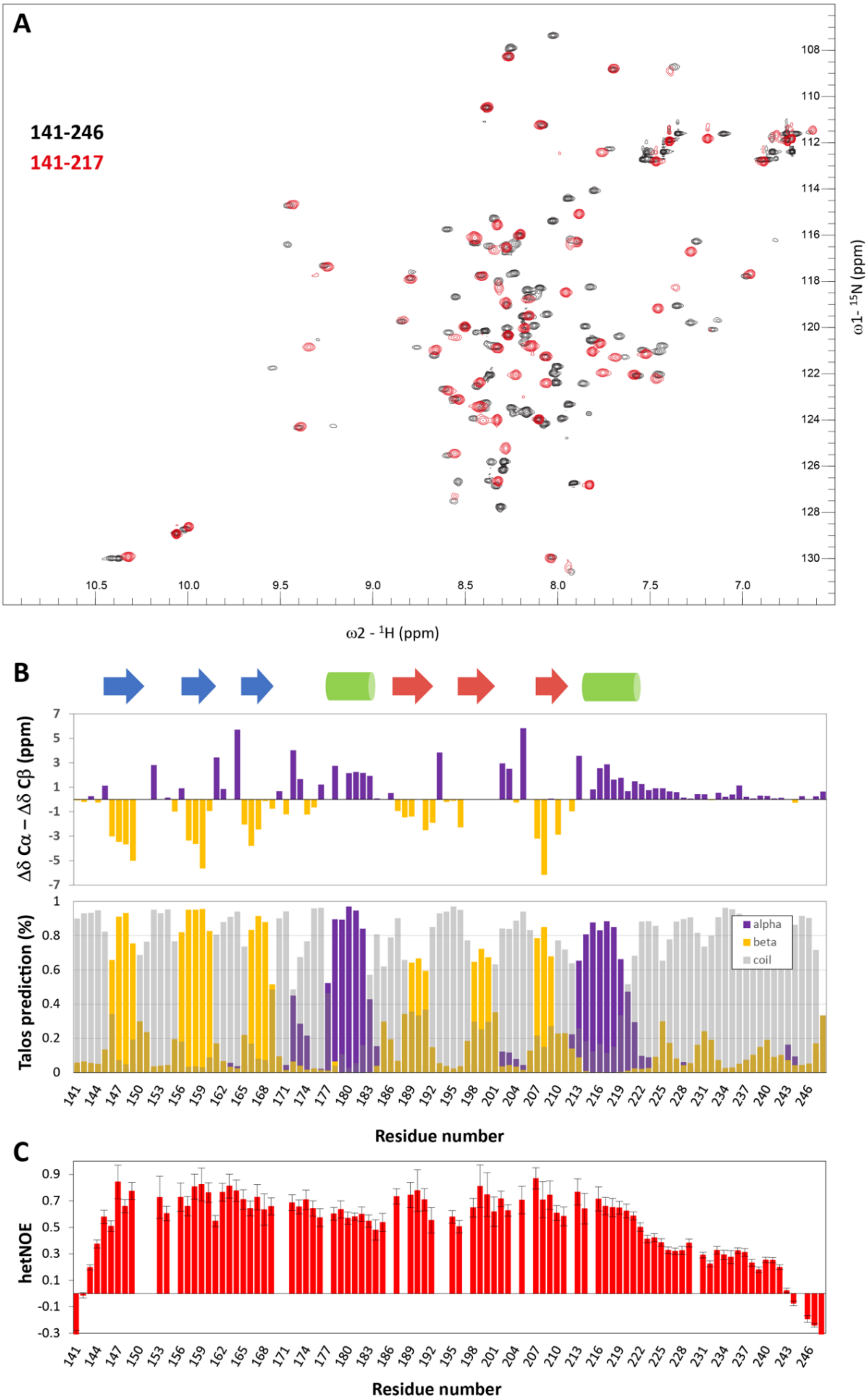

Supplementary Figure 2. NMR analysis of an extended version of the WW domain construct. A) ^{1}H-^{15}N HSQC spectra of the original construct ending in 217 (red) and the extension up to residue 248 (black), showing several chemical shift differences of the common peaks and the appearance of a number of new peaks in the ^{1}H region between 7 and 8 ppm. B) Chemical shift analysis of

the extended version: comparison of Cα and Cβ values versus the random coil chemical shift for each residue (up) and prediction made by Talos N software of the probability of secondary structure propensity after evaluation of the assigned chemical values for the extended version (down). C) $\{^{1}H\}$-^{15}N heteronuclear NOE values measured for the extended construct (red bars) and propagated experimental error calculated by CCPN analysis V2.5 (error lines). Source data are provided as a Source Data file.

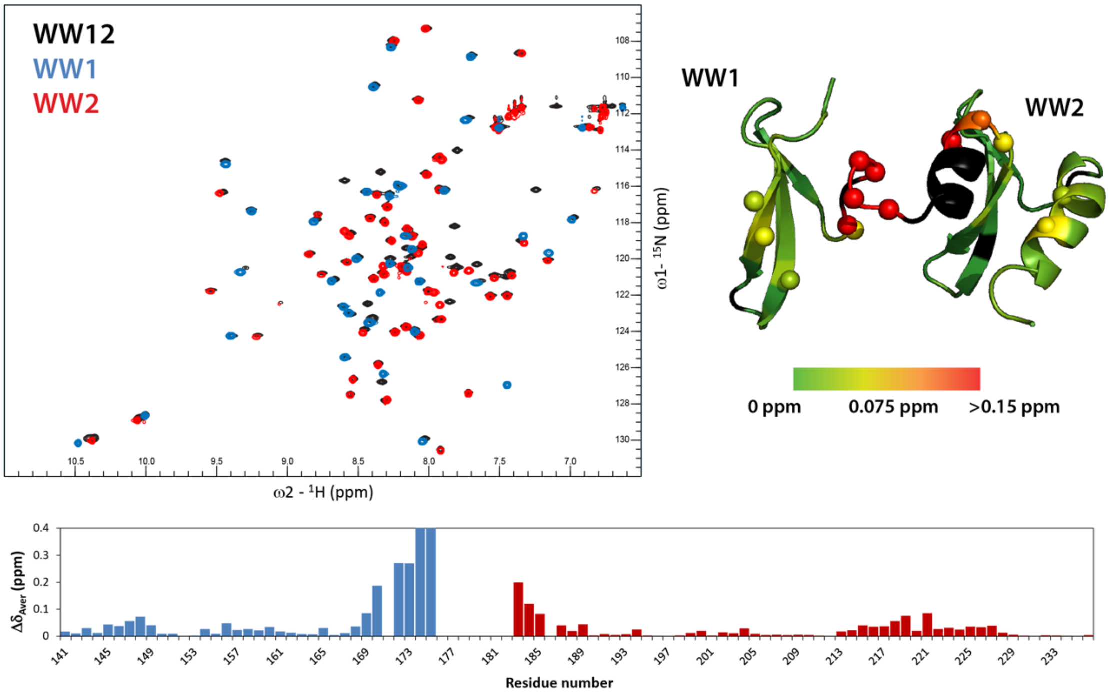

Supplementary Figure 3. WW1 and WW2 fold independently of each other. ^{1}H-^{15}N HSQC spectra comparison of WW12 (141-236, black), WW1 (141-176, blue) and WW2 (184-236, red) constructs (upper left). Chemical shift perturbation analysis is plotted (down) and mapped on the new structure (this work, upper right) according to the difference values (from green to red – no data in black, balls represent those residues with higher than 0.05 ppm difference). Source data are provided as a Source Data file.

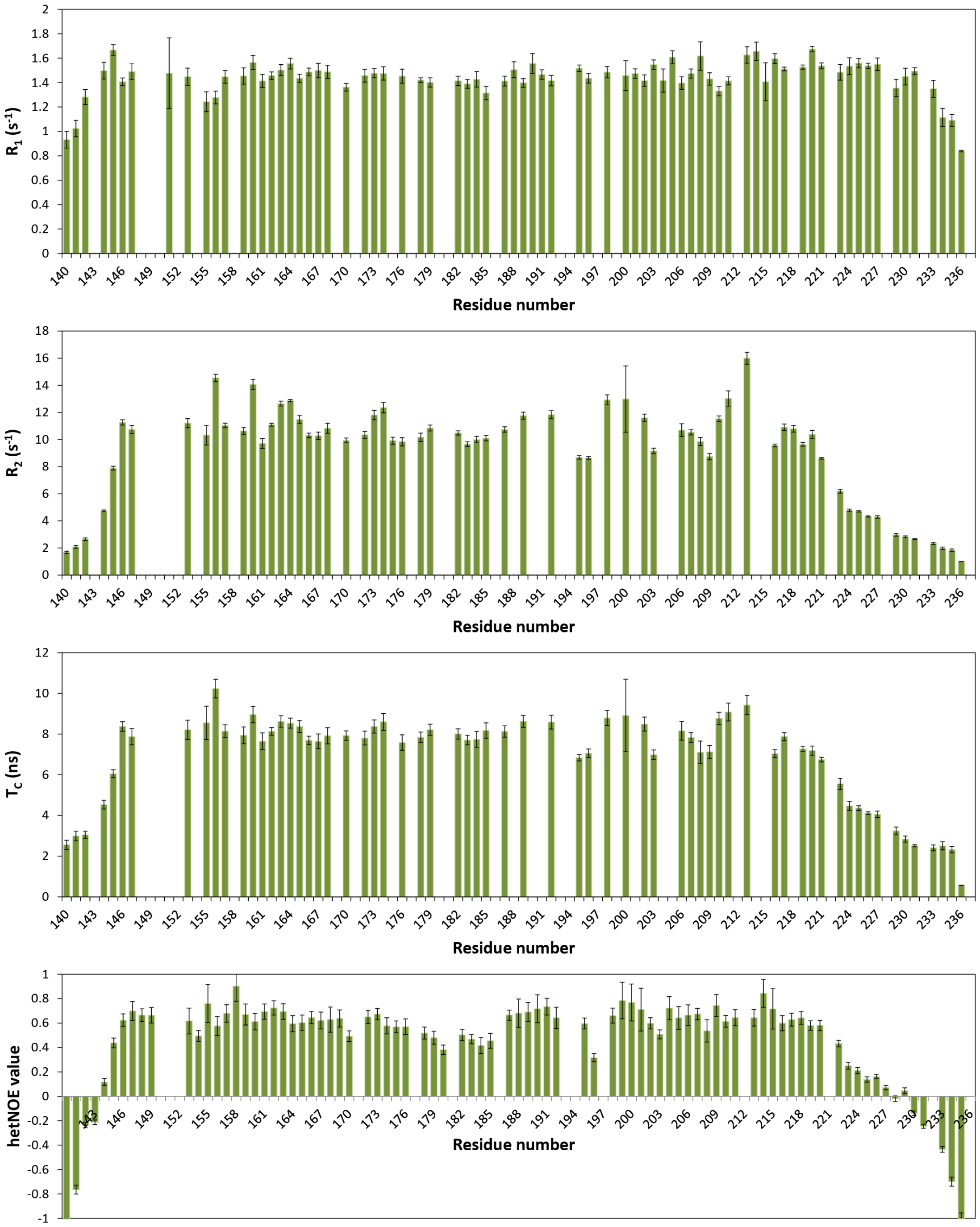

Supplementary Figure 4. NMR relaxation analysis of WW12. Measurement of ^{15}N R_1 and R_2 relaxation rates (up) for each residue in the construct, then the calculation of the correlation time τ_c derived from the ratio between R_2 and R_1 values, and the {^{1}H}-^{15}N heteronuclear NOE values (bottom). Error bars indicate the fitting error calculated using CCPN2 analysis V2.5 software (*R*1, *R*2), the propagated error for the correlation time, based on *R*1/R2 errors, and the propagated experimental error calculated by CCPN analysis V2.5. Source data are provided as a Source Data file.

A

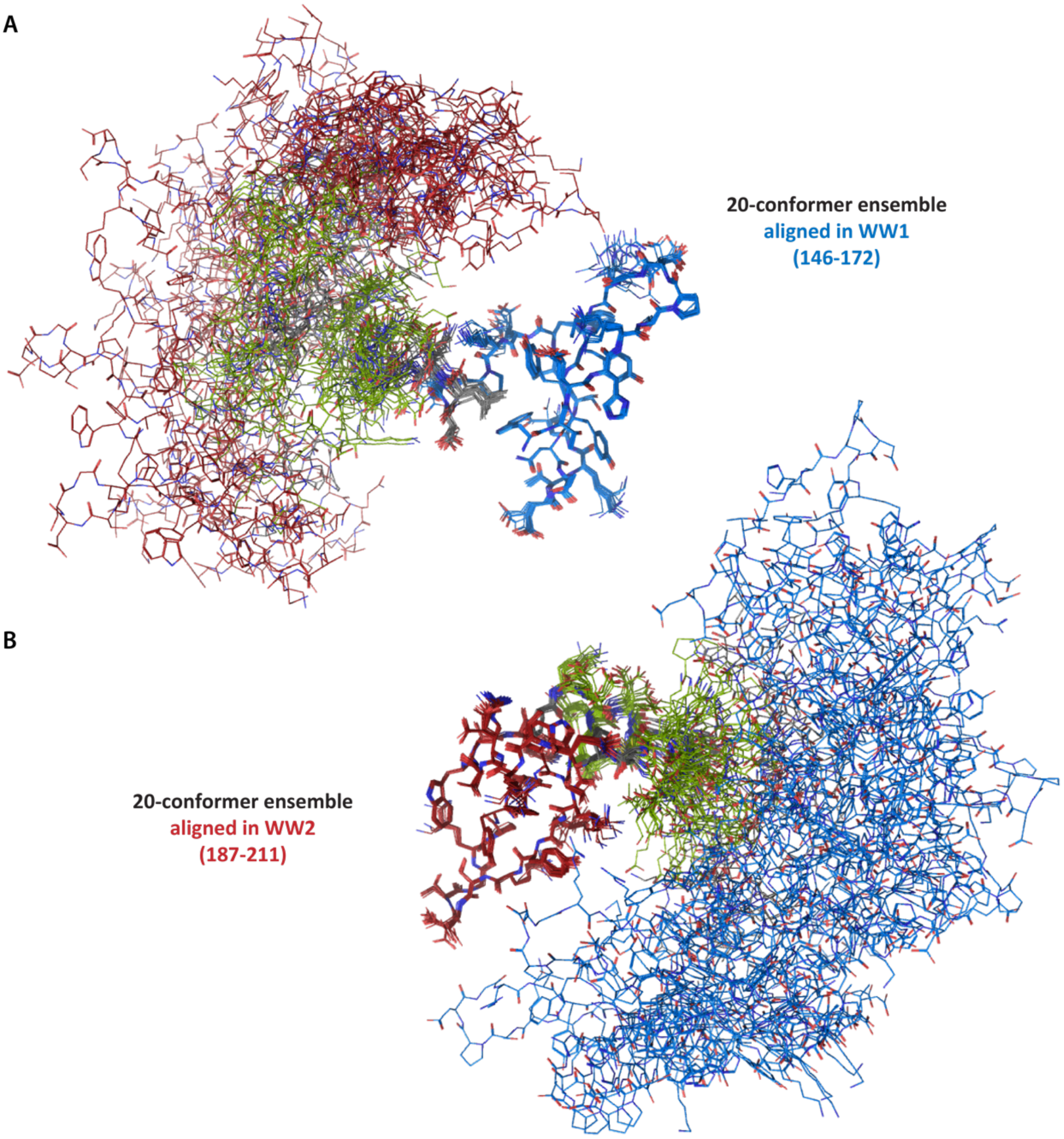

Supplementary Figure 5. WW domains present different orientations along the NMR-calculated ensemble. Line representation of the 20-conformer ensemble of PRPF40A WW12 region, aligned in the first WW domain (**A**) and in the second WW domain (**B**).

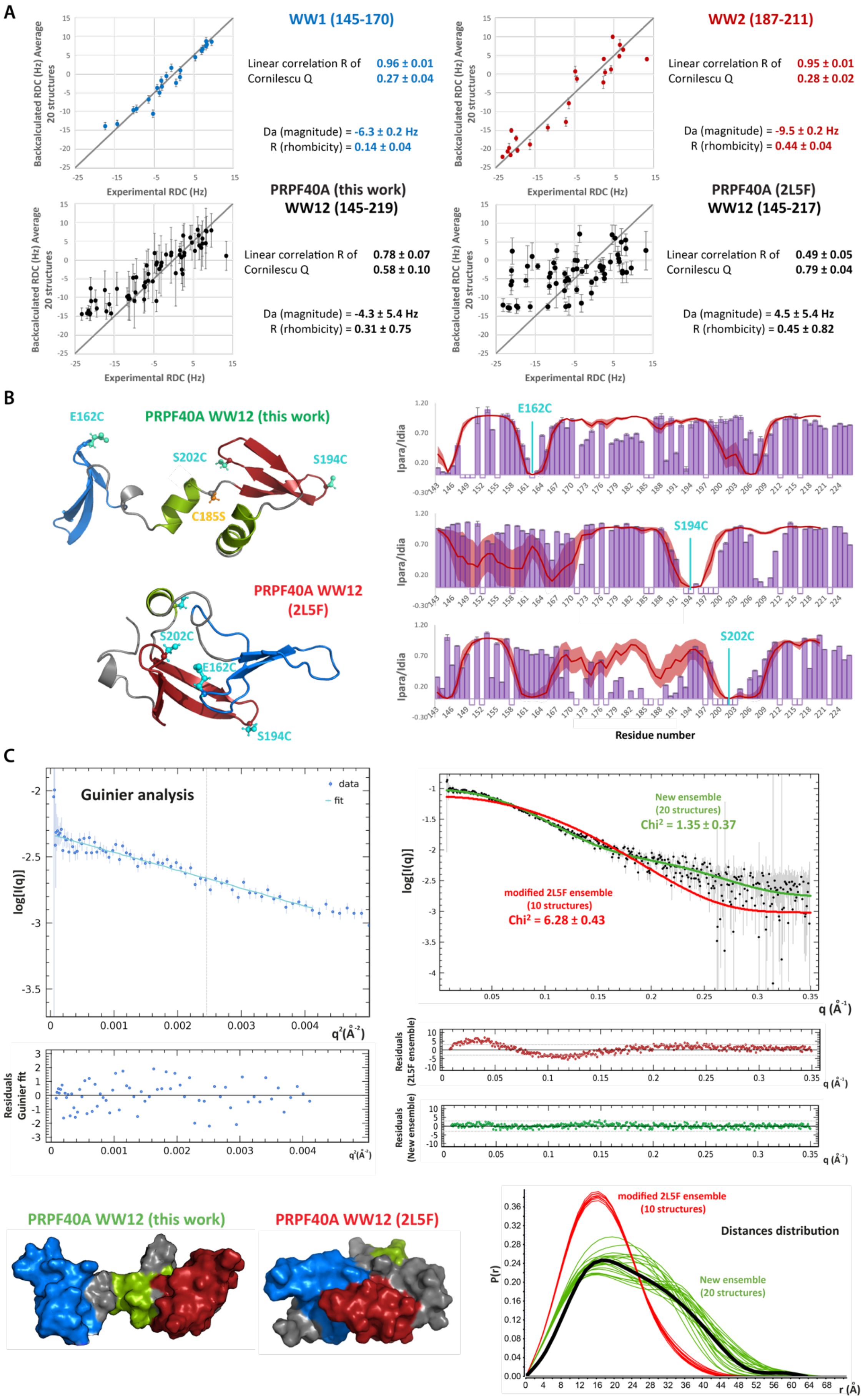

A
WW1 (145-170)
Linear correlation R of 0.96 ± 0.01
Cornilescu Q 0.27 ± 0.04
Da (magnitude) = -6.3 ± 0.2 Hz
R (rhombicity) = 0.14 ± 0.04
WW2 (187-211)
Linear correlation R of 0.95 ± 0.01
Cornilescu Q 0.28 ± 0.02
Da (magnitude) = -9.5 ± 0.2 Hz
R (rhombicity) = 0.44 ± 0.04
PRPF40A (this work)
WW12 (145-219)
Linear correlation R of 0.78 ± 0.07
Cornilescu Q 0.58 ± 0.10
Da (magnitude) = -4.3 ± 5.4 Hz
R (rhombicity) = 0.31 ± 0.75
PRPF40A (2L5F)
WW12 (145-217)
Linear correlation R of 0.49 ± 0.05
Cornilescu Q 0.79 ± 0.04
Da (magnitude) = 4.5 ± 5.4 Hz
R (rhombicity) = 0.45 ± 0.82
Backcalculated RDC (Hz) Average 20 structures
Experimental RDC (Hz)
B
E162C
PRPF40A WW12 (this work)
S202C
S194C
C185S
PRPF40A WW12 (2L5F)
Ipara/Idia
Residue number
C
Guinier analysis
data
fit
log[I(q)]
q²(Å⁻²)
Residuals Guinier fit
New ensemble (20 structures)
Chi² = 1.35 ± 0.37
modified 2L5F ensemble (10 structures)
Chi² = 6.28 ± 0.43
q (Å⁻¹)
Residuals (2L5F ensemble)
Residuals (New ensemble)
PRPF40A WW12 (this work)
PRPF40A WW12 (2L5F)
modified 2L5F ensemble (10 structures)
Distances distribution
New ensemble (20 structures)
P(r)
r (Å)

Supplementary Figure 6. Structural validation of PRPF40A WW12 ensemble and comparison with previously reported structure. (**A**) Evaluation of the residual dipolar coupling data for the backbone amino signals. Pales best-fit to the PRPF40A WW1 (up left), WW2 (up right) and WW12 (down left) of the PRPF40A structure obtained in this work and compared with the old WW12 truncated structure (2L5F, down right). Dots indicate averaged calculated RDC value for each residue among the 20/10 structures in the ensemble vs the experimental value; the error bars indicate the standard deviation between the calculated values for each member of the ensemble. Good agreement is obtained for the individual domains but with different tensor parameters, reflecting different partial alignment of both domains due to the presence of dynamics between WW domains in solution. (**B**) Cartoon representation of the new and old structures of WW12 PRPF40A indicating the positions of the mutated residues for IPSL labelling (left). PRE data for the three mutants (right), same as Figure 1B, but in this case compared to the expected distances in the old, truncated structure: purple bar charts representing the experimental intensity ratios for each amine signal in the ^{1}H-^{15}N HSQCs between the paramagnetic and diamagnetic states of the IPSL-labeled proteins; propagated experimental errors are shown as gray bars. The red line and the shadow represent the calculated intensity ratios based on the old structure of the PRPF40A WW12 and the deviation of the value in the 10-conformer ensemble. Negative values indicate no data for those residues. Data does not agree with this structure due to the lack of interdomain short distances. (**C**) SAXS analysis of PRPF40A. Upper part: Guinier analysis of the data (left) and fitting to the two ensemble structures (right): the old NMR ensemble of WW12 (red) and the new NMR ensemble (green); residuals for each fit are shown below the curve. Error bars in both graphs indicate SAXS measurement errors. Lower part: the overall shape of both structures of WW12 clearly shows the more extended conformation in the new structure compared to the compact structure of the truncated version (left). This compact particle shape (red) does not agree with the distance distribution plot derived from the SAXS curve, while the new ensemble (green) shows a similar bimodal distribution, characteristic of dumbbell-like particles (right). Notice that the fitting to the truncated structure was done with extended pdb files that incorporate the C-terminal helix and all necessary residues to cover the construct used in the SAXS acquisition. Source data are provided as a Source Data file.

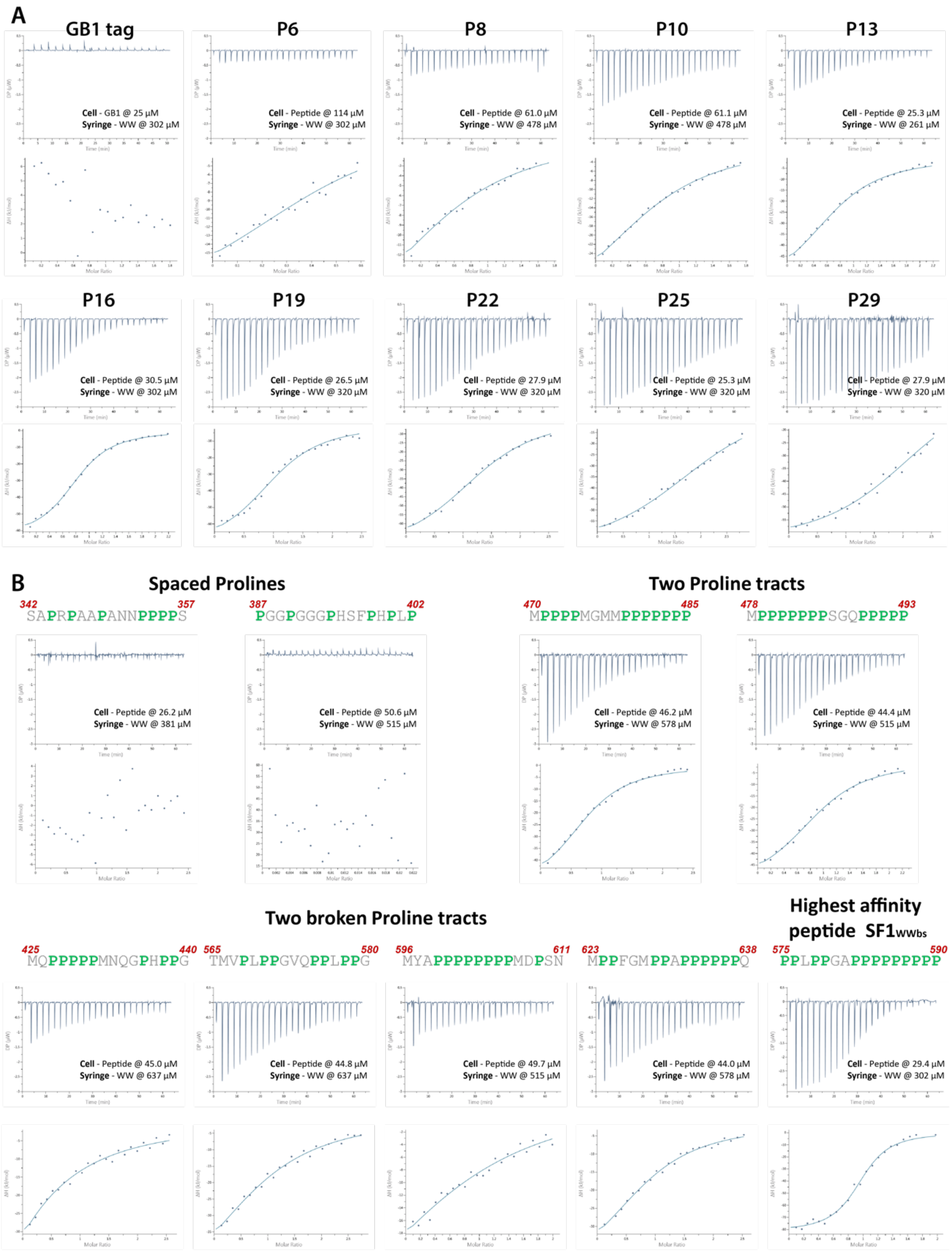

Supplementary Figure 7. Isothermal Titration Calorimetry conditions, curves and fitting for the interactions of WW12 of PRPF40A with different peptides. Only one titration is presented from the at least 2 repeated experiments. (**A**) Titrations with GB1-polyproline peptides (6-29) and the blank (using only GB1 tag). (**B**) Titrations with different GB1-SF1 16mer peptides, the peptide sequences appear in the top and correspond to the following residue numbers of the isoform 1 of SF1: 342-357, 387-402, 470-485, 478-493, 425-440, 565-580, 596-611, 623-638, 575-590. Source data are deposited in Zenodo repository.

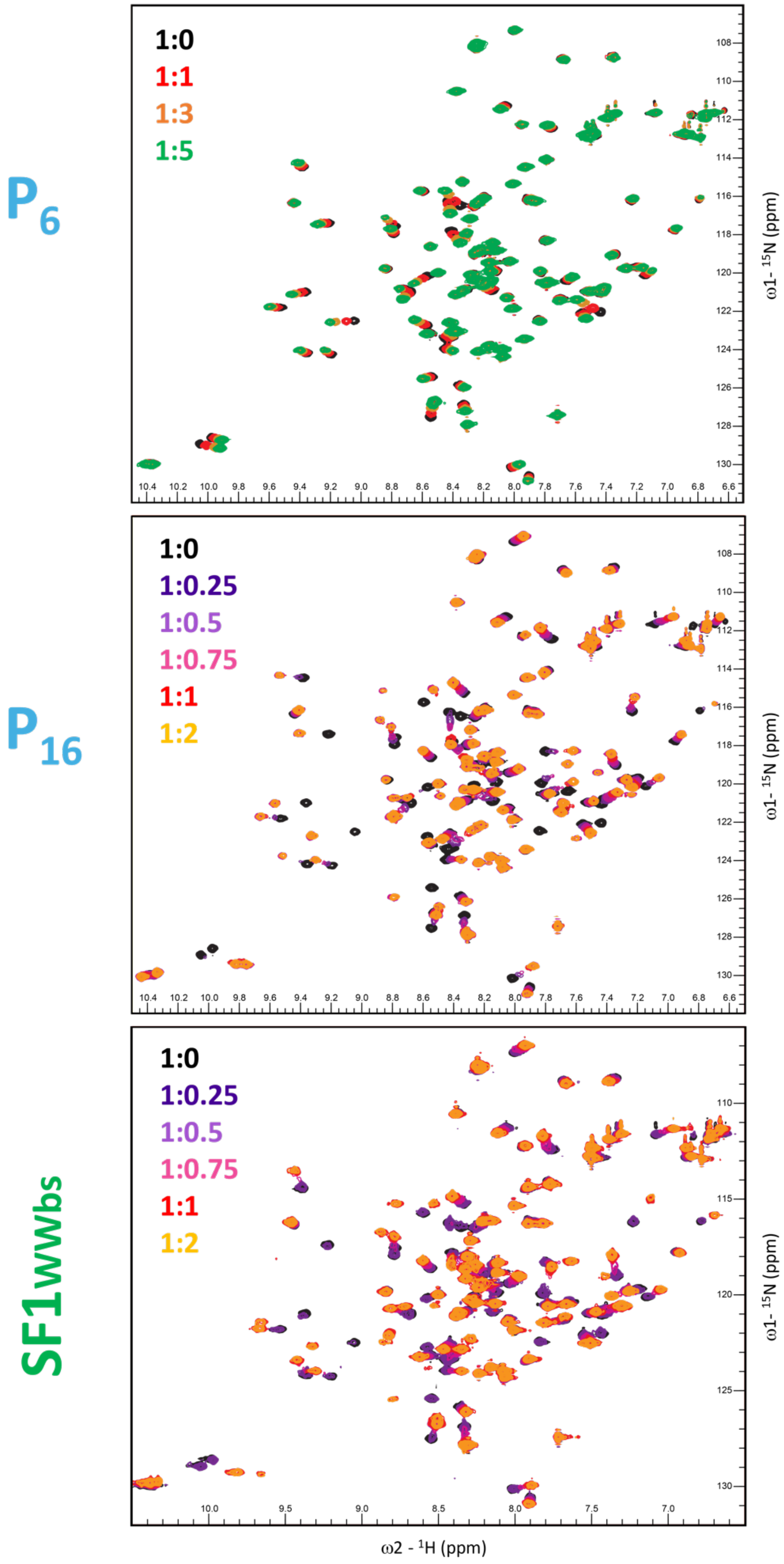

Supplementary Figure 8. ^{1}H-^{15}N HSQC spectra showing the titration points of different peptides (P6 – up, P16 – middle and $SF1_{WWbs}$ – down) to PRPF40A WW12 construct.

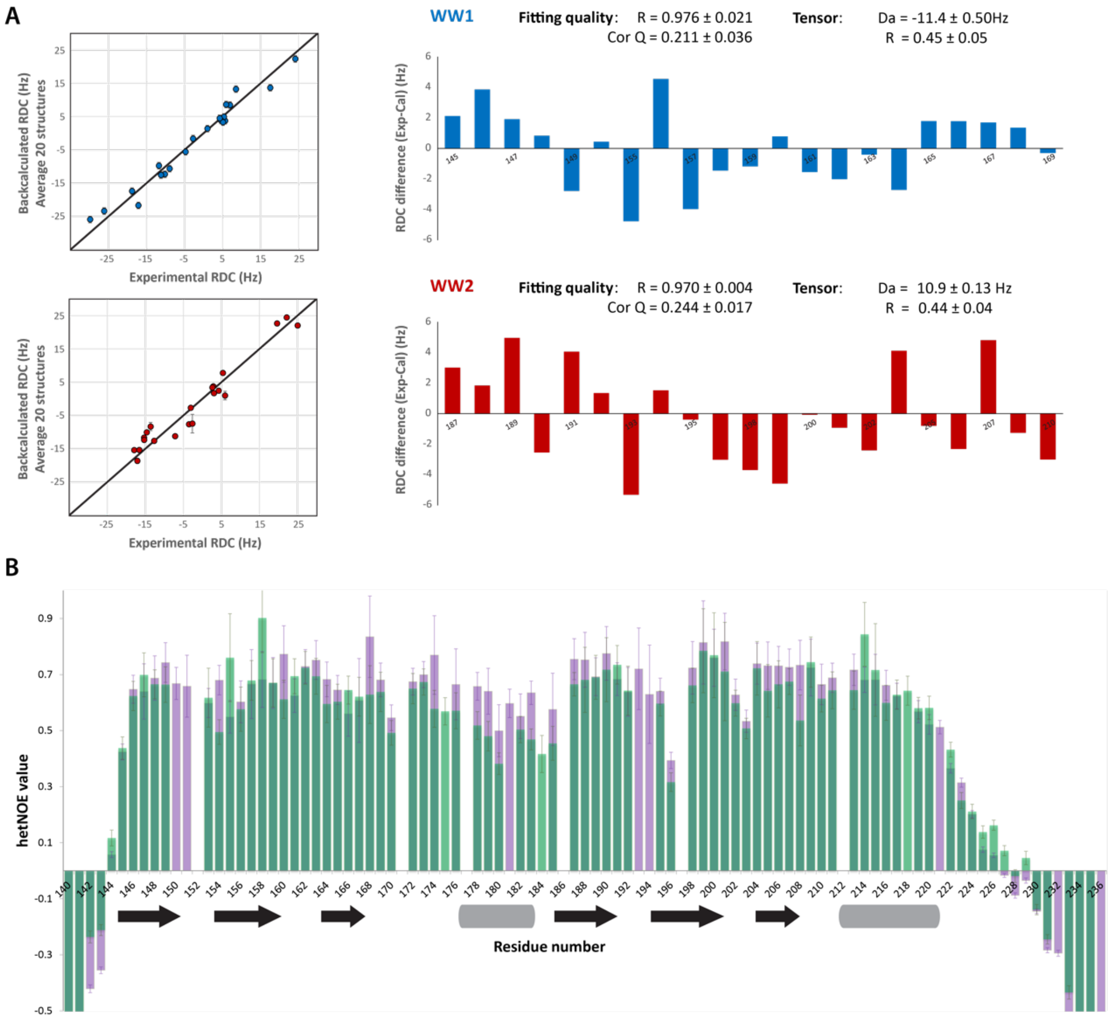

Supplementary Figure 9. The WW12 tandem conformation shows less dynamics in the complex with SF1$_{WWbs}$ than in the apo state. (**A**) RDC data of the complex fitted to the WW1 or WW2 structures (apo). Dots indicate averaged calculated RDC value for each residue among the 20 structures in the ensemble vs the experimental value; the error bars indicate the standard deviation between the calculated values for each member of the ensemble. The fitting is good and the tensor parameters extracted for each domain are closer than in the apo state (Supplementary figure 5) meaning that they share the partial alignment orientation respect to the magnetic field. (**B**) {^{1}H}-^{15}N heteronuclear NOE values of the WW12 in apo (green) and in bound states (purple). Error lines indicate the propagated experimental error calculated by CCPN analysis V2.5. The region between resides 176-186 (linker helix) shows higher NOE values in the complex indicating that they experience less dynamics than in the apo version. Source data are provided as a Source Data file.

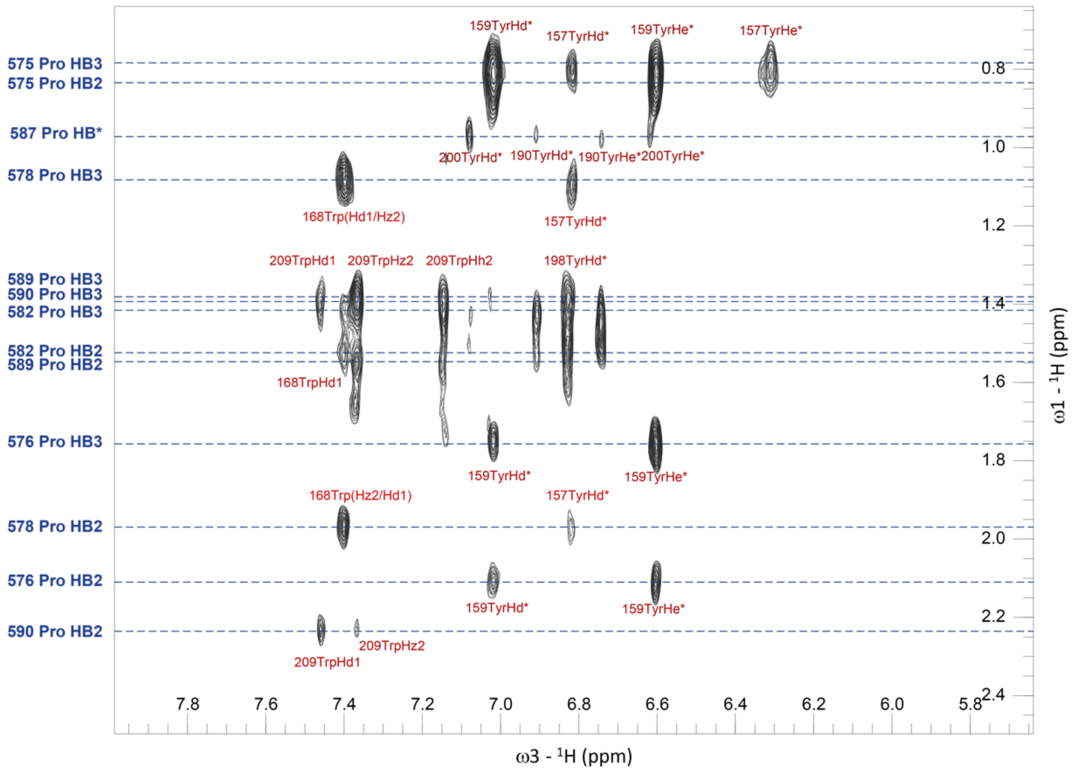

Supplementary Figure 10. Example of intermolecular NOESY cross-peak assignment. Overlay of 3D ^{13}C-edited NOESY planes corresponding to the proline Cβ region (ω_2 region – ^{13}C: 29.1 to 33.4 ppm) acquired for a sample of the SF1$_{WWbs}$/PRPF40A WW12 complex where the SF1 peptide was ^{13}C labeled. Pro Hβ protons of SF1 give NOE cross-peaks to several H aromatic resonances from the WW domains of PRPF40A. Note that the peptide does not contain any aromatic residue, thus allowing a clear and unambiguous assignment of several intermolecular NOEs.

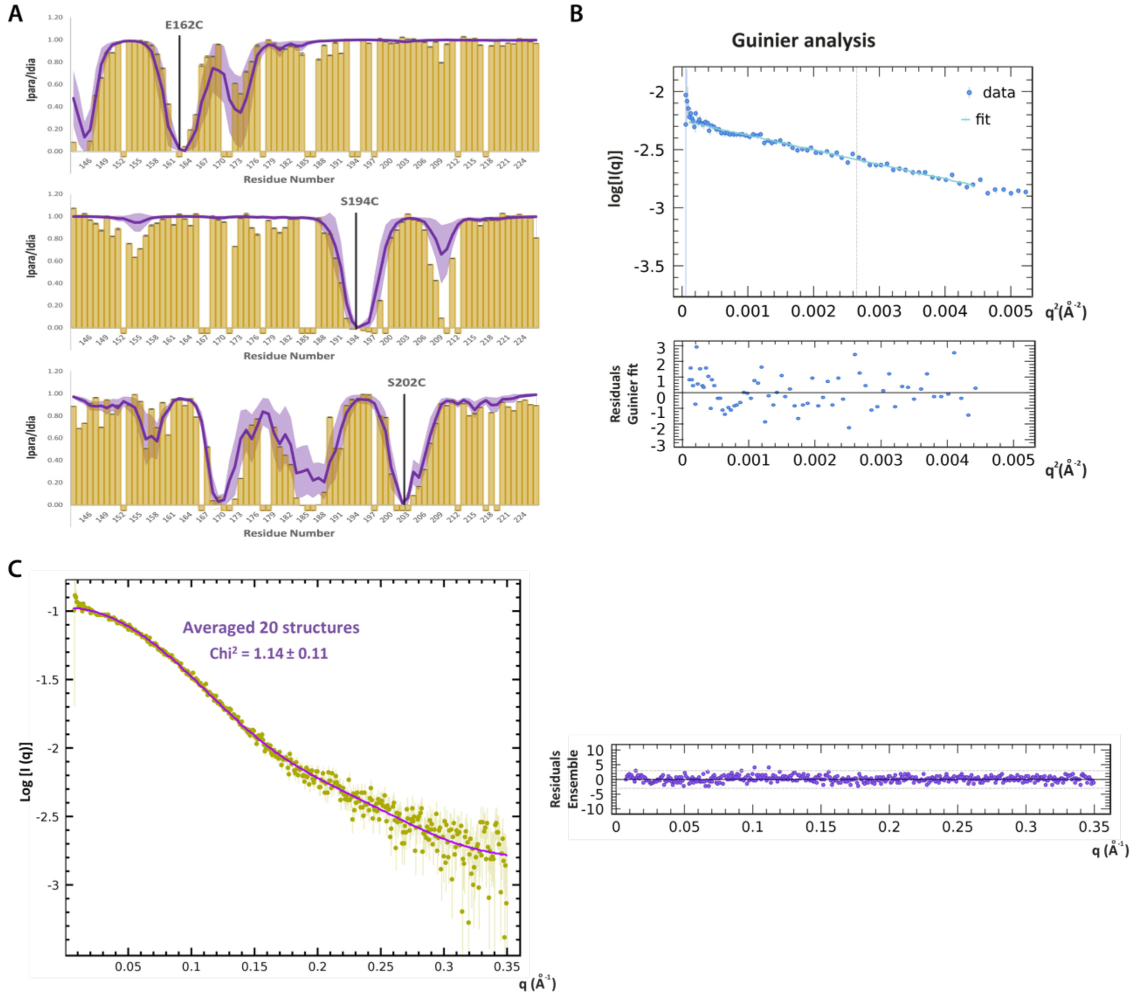

Supplementary Figure 11. Structural validation of the PRPF40A WW12 -$SF1_{WWbs}$ complex structure. (**A**) Bar charts representing the intensity ratios for each amine signal in the ^{1}H-^{15}N HSQCs between the paramagnetic and diamagnetic states of the IPSL labelled complexes (yellow bars) and the propagated experimental error (error lines). Same three different positions were selected for labelling as for the apo WW12: E162, S194 and S202. The purple line and shadow represent the calculated intensity ratios based on the structure of the complex and the deviation of the value in the 20-conformer ensemble. (**B** & **C**) SAXS analysis of PRPF40A WW12 bound to $SF1_{WWbs}$. Guinier analysis of the SAXS curve for the complex with the residuals of the fit below (**B**). Fitting of the averaged ensemble of the complex (purple line) to the SAXS data in yellow dots (left); residuals for the fit are shown on the right (**C**). In all SAXS curves (**B & C**), dots indicate scattering intensity values and lines the measurement errors. Source data are provided as a Source Data file.

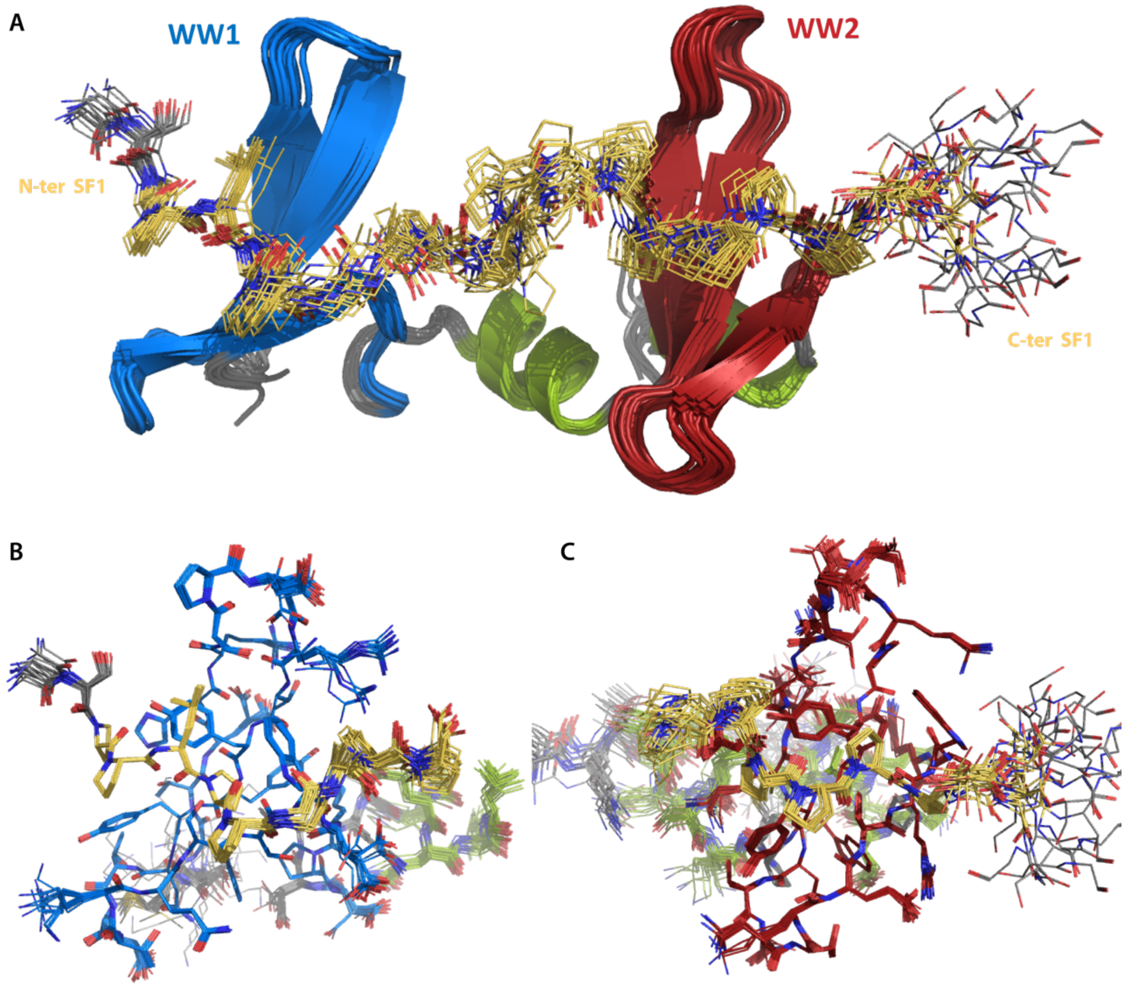

Supplementary Figure 12. Structural ensemble of the WW12 of PRPF40A in complex with SF1$_{WWbs}$ peptide. Fitting to the complete tandem structure (**A**) or to the WW1 (**B**) or WW2 domains (**C**).

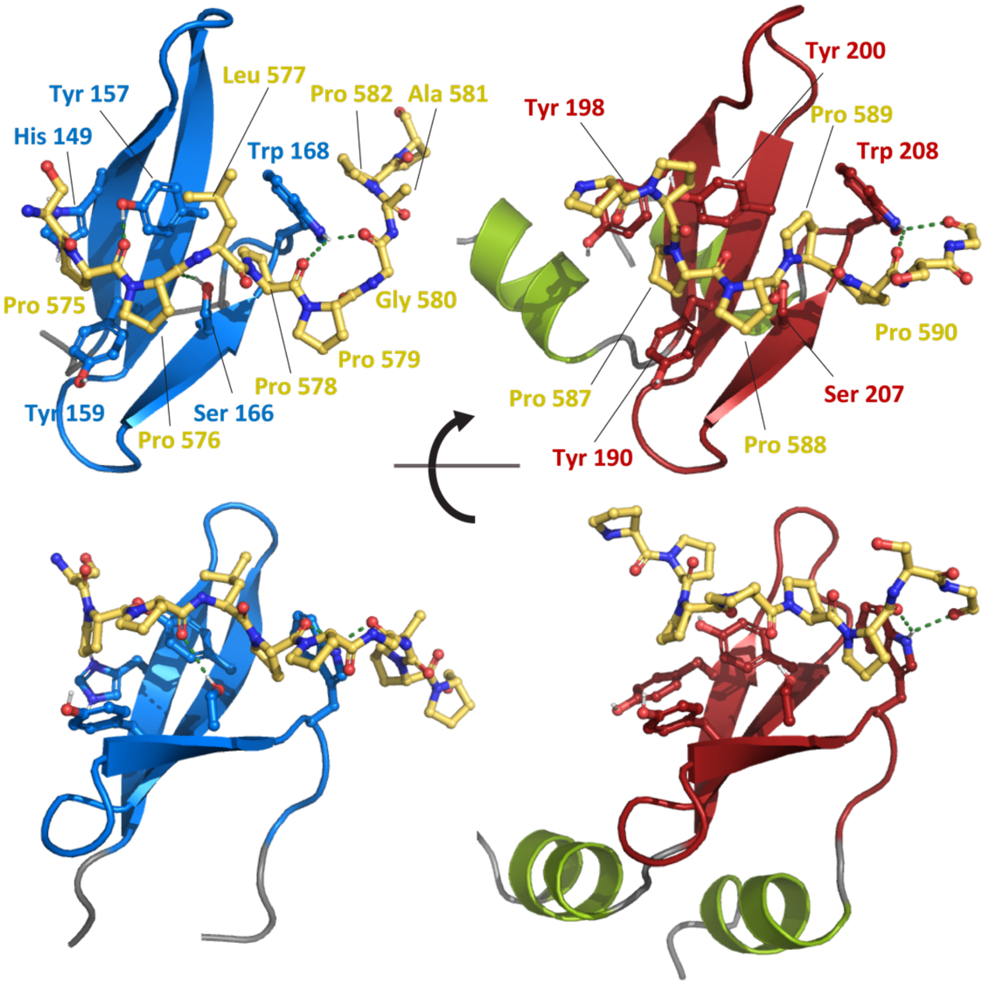

Supplementary Figure 13. Depicted view of the recognition of the proline rich peptides (in yellow sticks) by the WW1 and WW2 domains (in cartoon representation), showing the key residues involved in binding and SF1$_{WWbs}$ pepetide as sticks. Key hydrogen bonds/polar contacts are shown as green dashed lines.

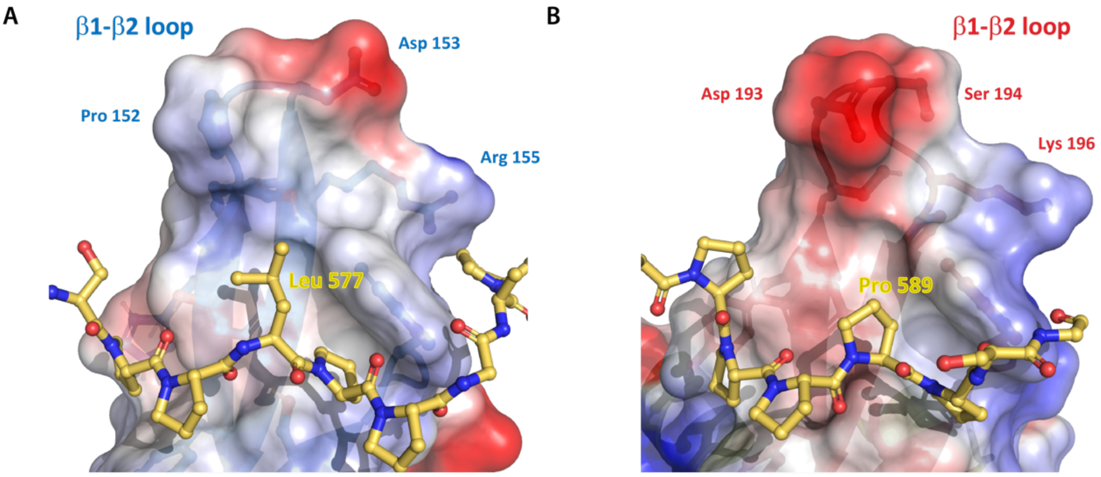

C

```
PRPF40A
148 EHKSPDGRTYYYN 160
189 EYKSDSGKPYYYN 201
    *.**--*.-****
```

```
SF1
GSPPLPPGAPPPPPPPPPSG
 575              590
```

Supplementary Figure 14. Differences between WW1 (**A**) and WW2 (**B**) recognition of SF1WWbs peptide in the β1-β2 region. (**C**) Sequence differences of the β1- β2 loop between WW1 and WW2 of PRPF40A and SF1WWbs peptide sequence, highlighting those residues recognized by WW1 (blue) and by WW2 (red).

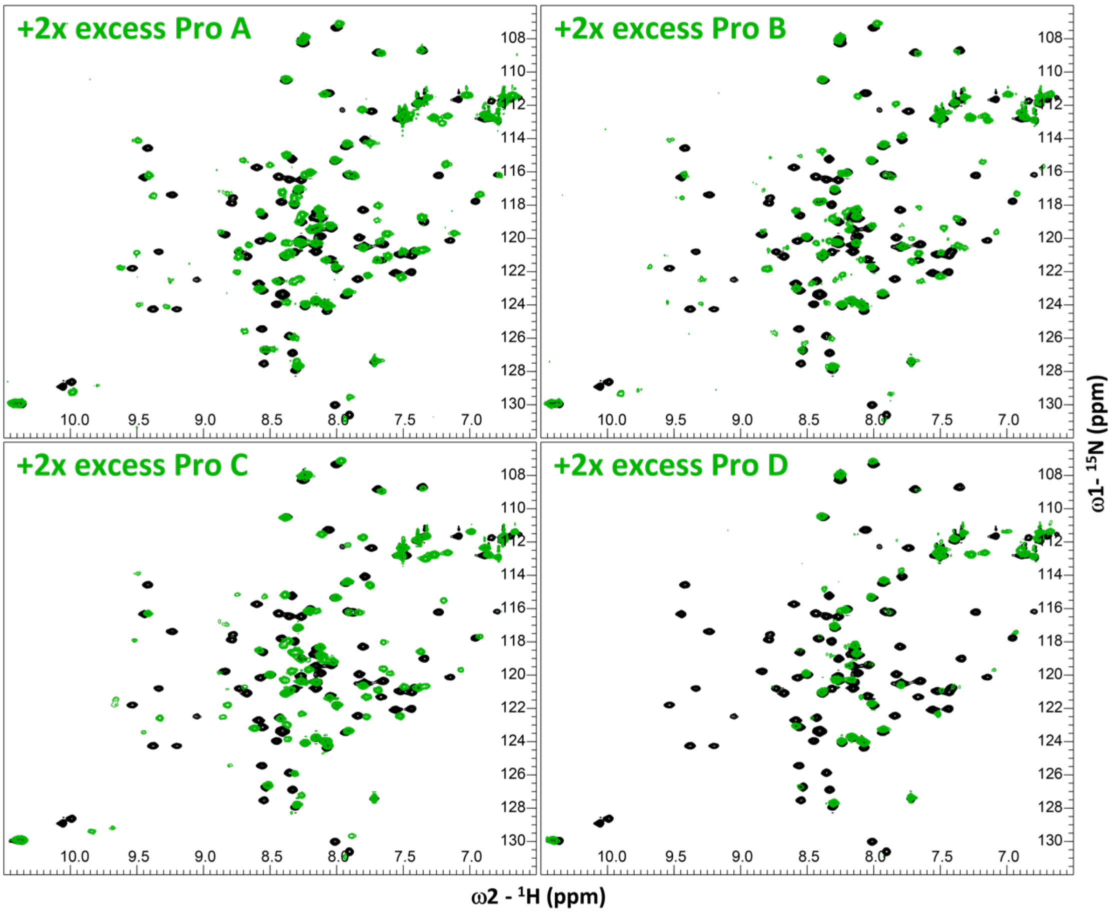

Supplementary Figure 15. ^{1}H-^{15}N HSQC spectra overlay of free PRPF40A WW tandem (black) and titrated with two-fold molar excess of the shorter SF1 constructs Pro A to Pro D (green).

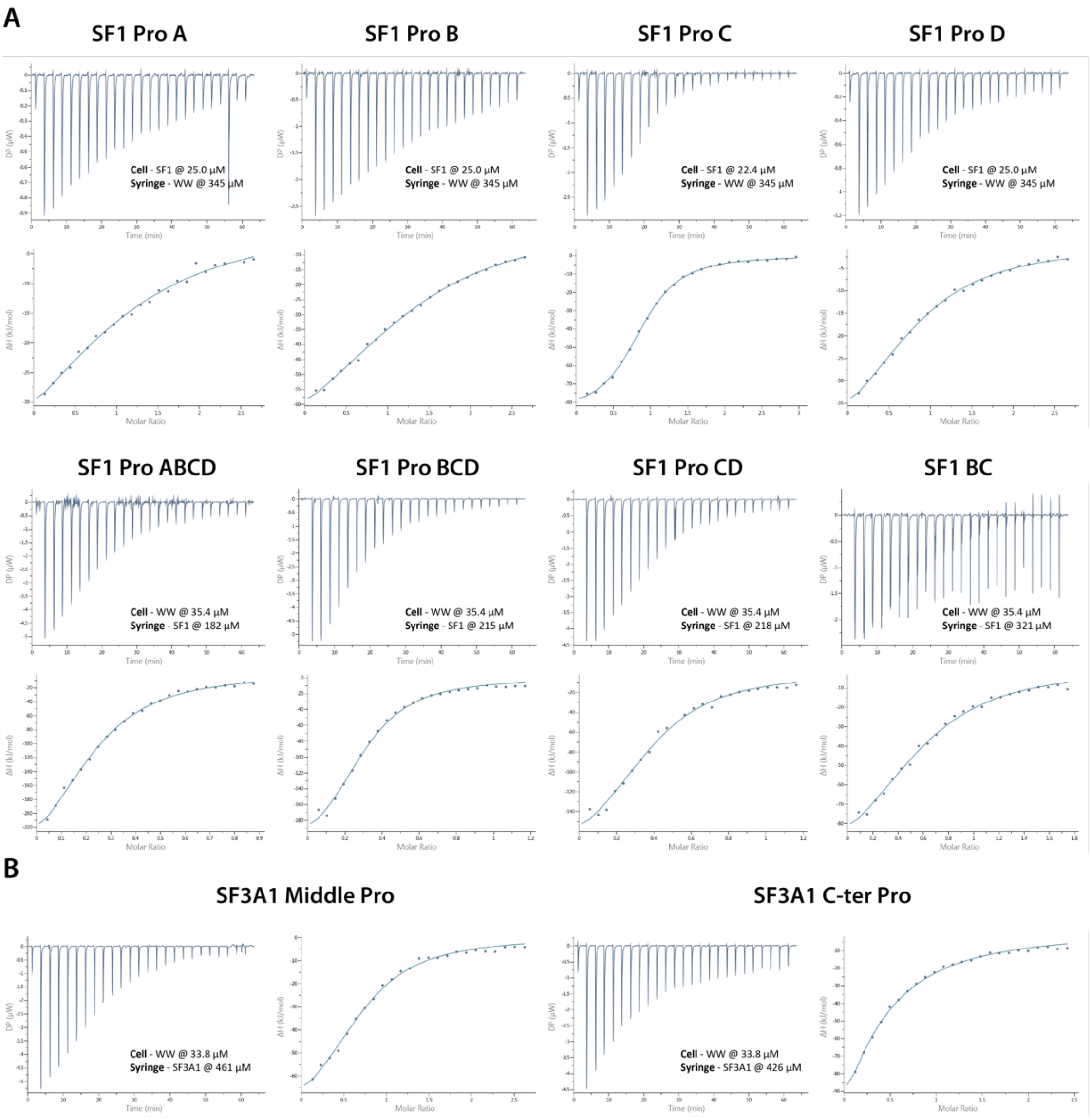

Supplementary Figure 16. Isothermal Titration Calorimetry conditions, curves and fitting for the interactions of WW12 of PRPF40A with SF1 (**A**) and SF3A1 different constructs (**B**). Source data are deposited in Zenodo repository.

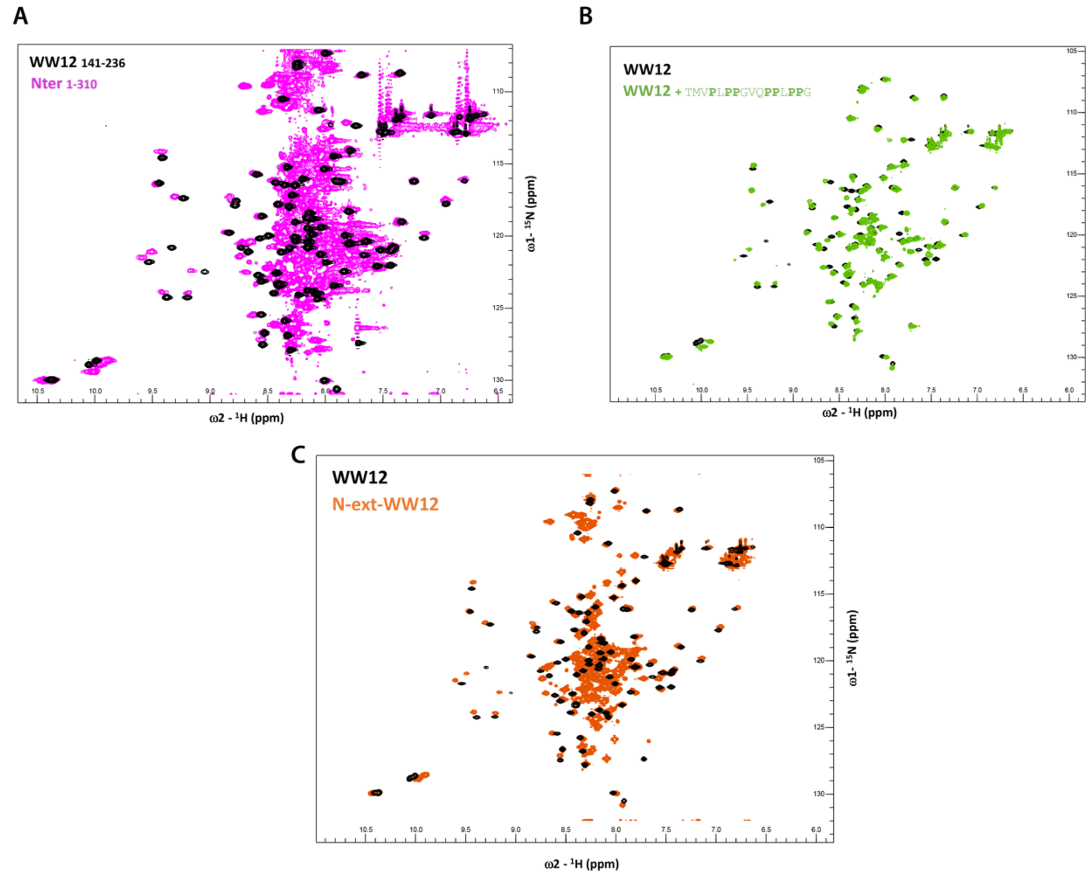

Supplementary Figure 17. The N-terminal region of PRPF40A interacts with the WW domains in a similar way as a Pro-rich peptide (**A**) ^{1}H-^{15}N HSQC spectra comparison of the WW12 construct of PRPF40A (black, 141-236) and the whole N-terminal region of the protein (magenta, 1-310). (**B**) ^{1}H-^{15}N HSQC spectra comparison of the WW12 construct free (black) and bound to a low-affinity peptide (green). Similar perturbation is observed in the WW12 peaks compared to the extended version (magenta in A). (**C**) ^{1}H-^{15}N HSQC spectra comparison of the WW12 construct (black) and the version N-terminally extended (orange, 56-236).

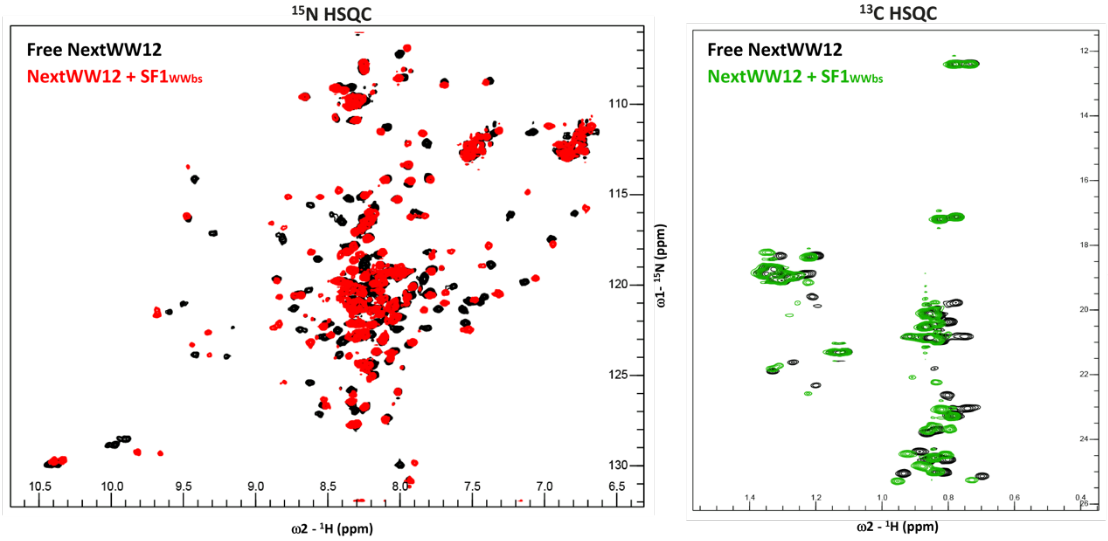

Supplementary Figure 18. NMR titration of the N-terminal extended WW12 construct with the high affinity peptide from SF1 ($SF1_{WWbs}$). ^{1}H-^{15}N HSQC (left) spectra overlay of the free (black) and bound (red at 1:2 molar ratio) and ^{1}H-^{13}C HSQC (right) spectra overlay of both states (free – black, bound – green) zoomed in the methyl region.

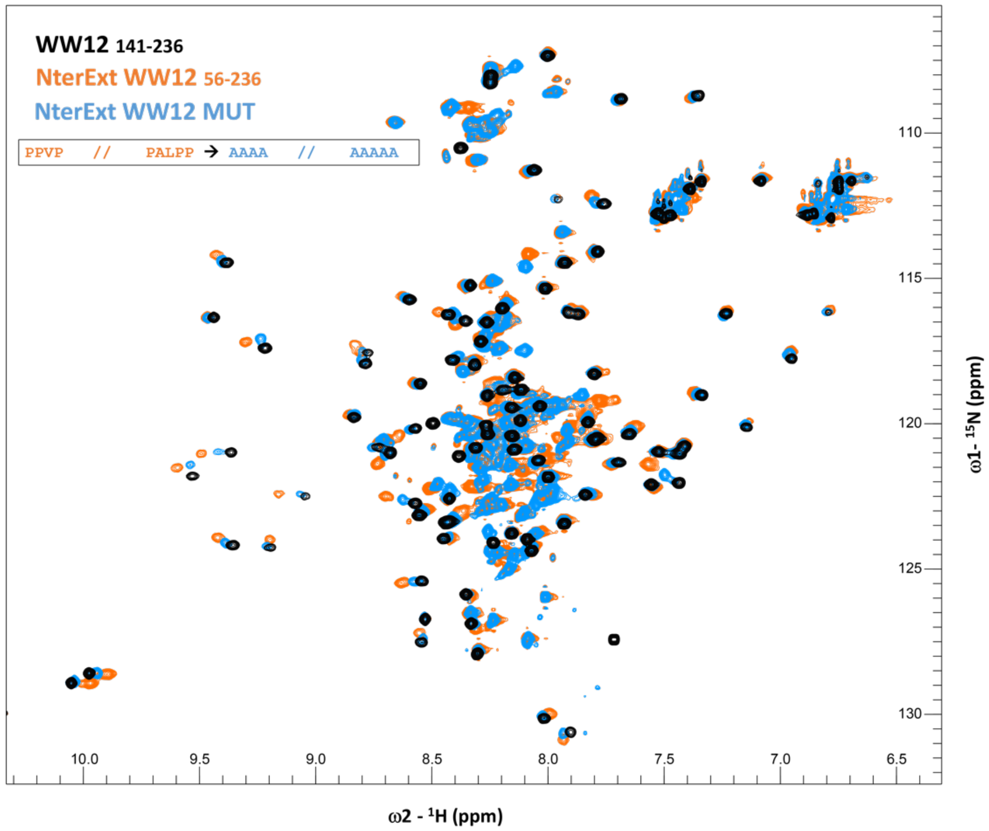

Supplementary Figure 19. The two motifs PPVP / PALPP at the N-terminal extension interact intermolecularly with the WW12 tandem. ^{1}H-^{15}N HSQC spectra comparison of WW12 tandem construct (black), N-terminal extended WW12 tandem (orange) and the mutant version (blue).

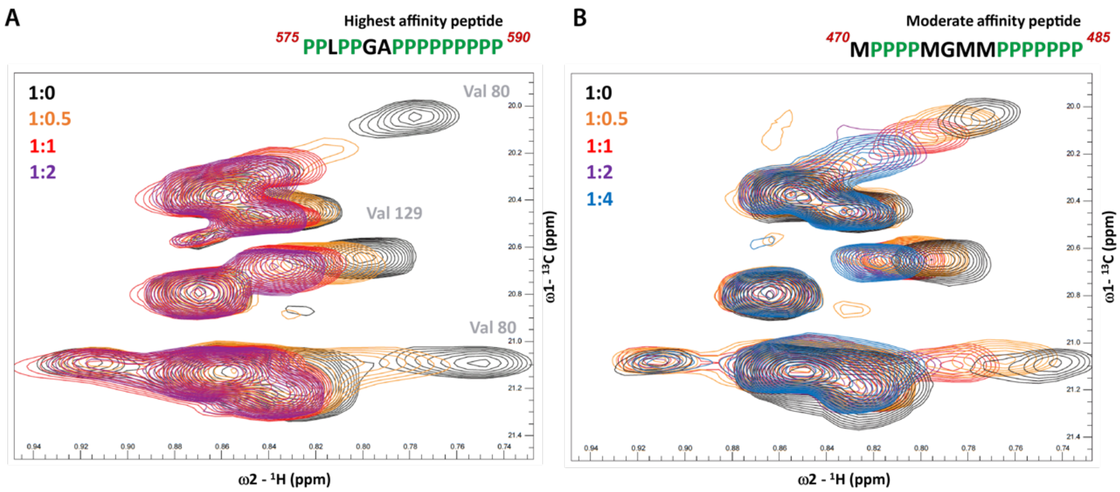

Supplementary Figure 20. The presence of the N-terminal extension tunes the selectivity of the WW domains for different proline-rich peptides. ^{1}H-^{15}N HSQC spectra overlay of two titrations to the N-terminally extended WW12 construct with the high-affinity peptide from SF1 ($SF1_{WWbs}$, left) and a moderate affinity peptide, also selected from SF1 (right).

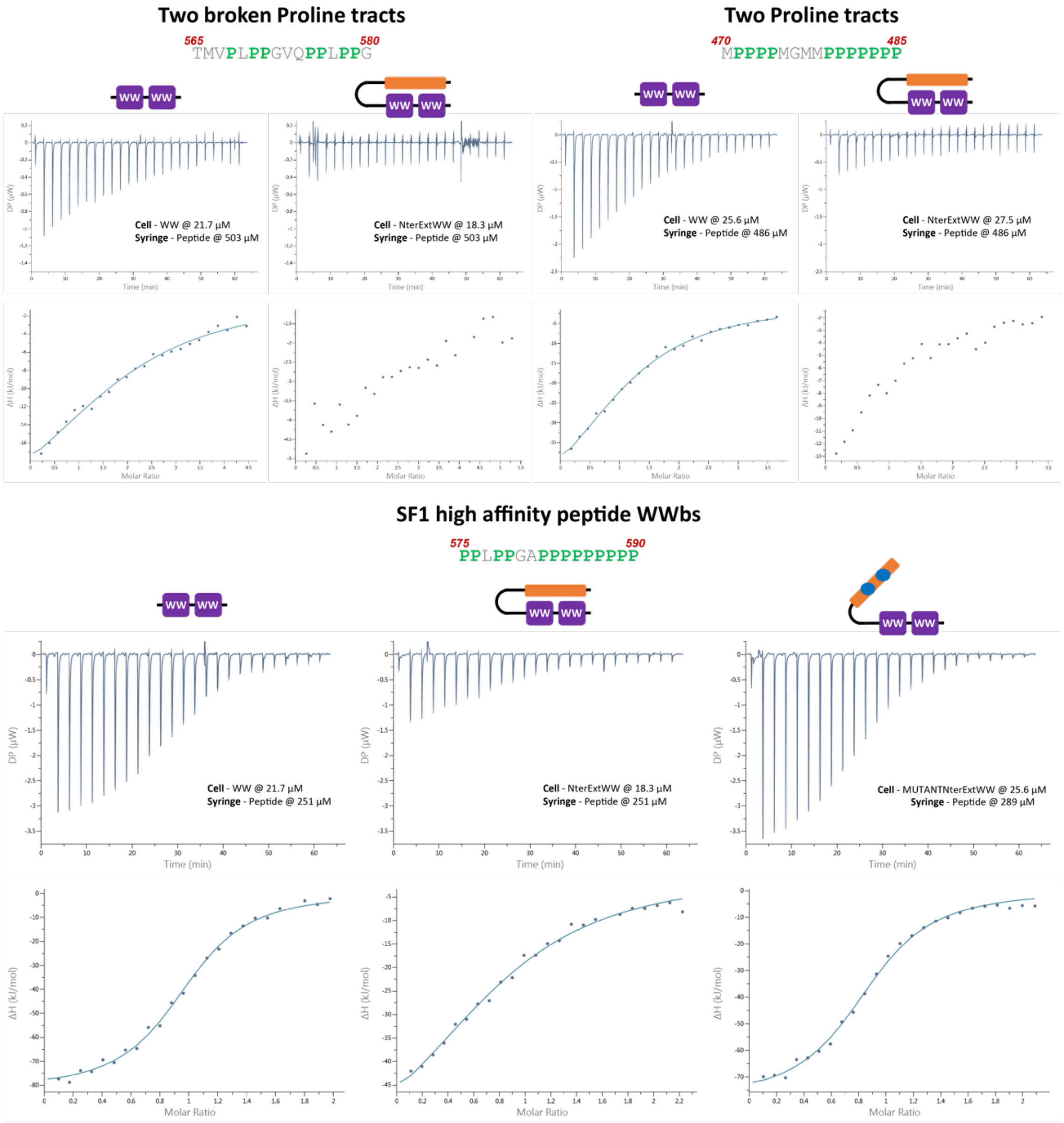

Supplementary Figure 21. Isothermal Titration Calorimetry conditions, curves and fitting for the interactions of WW12, N-ext-WW12 and mutant N-ext-WW12 constructs of PRPF40A with different SF1 peptides. Source data are deposited in Zenodo repository.

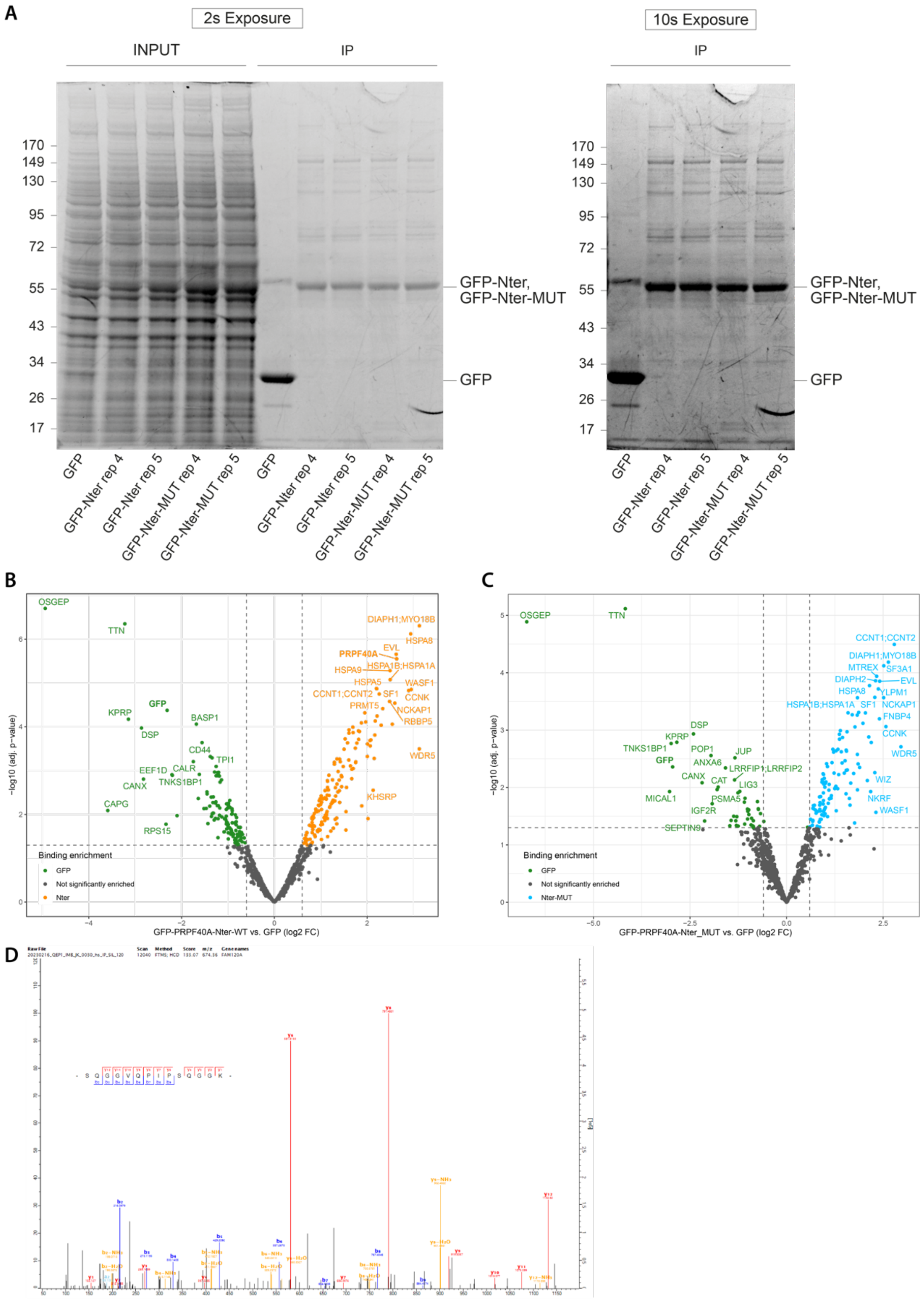

Supplementary Figure 22. Immunoprecipitation of the PRPF40A-Nter constructs and the following mass spectrometry analysis. (**A**) Polyacrylamide gels of the input and the immunoprecipitated proteins with GFP (negative control), GFP-PRPF40A-Nter-WT (GFP-Nter) and GFP-PRPF40-MUT (GFP-Nter-MUT) constructs. Some protein bands are more intense mutant lanes compared to the wild type lanes along the two repeats (biological repeat 4 and 5). Mass spectrometry quantification of the immunoprecipitated components for three additional biological replicates was performed. The volcano plots show the protein enrichment of PRPF40A-

Nter-WT (**B**) and PRPF40A-Nter-MUT (**C**) versus the GFP control. Significantly enriched proteins for GFP are shown in green, for PRPF40A-Nter-WT in orange and for PRPF40A-Nter-MUT, that lacks the intramolecular interaction between the proline-rich region and the WW-domains, is shown in blue. Significantly enriched proteins in the PRPF40A-Nter constructs are compared in Figure 5D. Minimal significant enrichment = log2(1.5) and p-value = 0.05. The p-values (two-sided) were determined by linear modeling using the limma package. (**D**) Annotated fragment spectrum of the unique peptide SQGGVQPIPSQGGK detected for protein FAM120A (Supplementary Table 5). Source data are provided as a Source Data file.

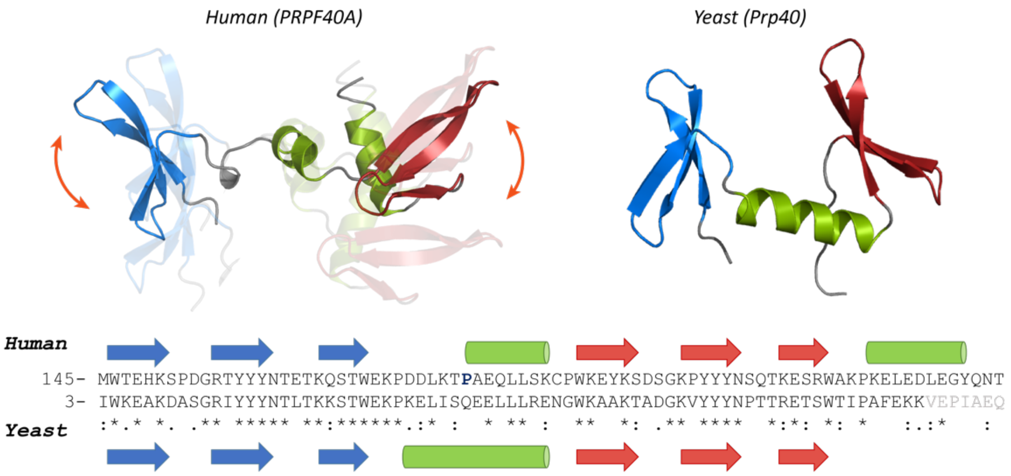

Supplementary Figure 23. Structure and sequence comparison of human PRPF40A (this work) and yeast Prp40 (PDB: 1O6W). Cartoon representation of the structures (top) of both homologs (human – left, yeast – right) and sequence alignment (down). Notice that the yeast version does not contain the C-terminal helix and the human homolog has a proline residue (bold) that disrupts the linker helix: there is an α-turn before the proline and the shorter helix after.

WW1 GB1_SF1 ProC titration

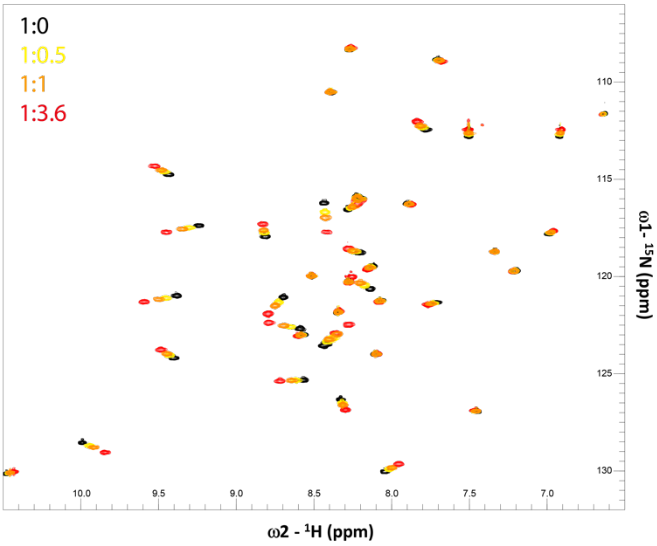

WW2 GB1_SF1 ProC titration

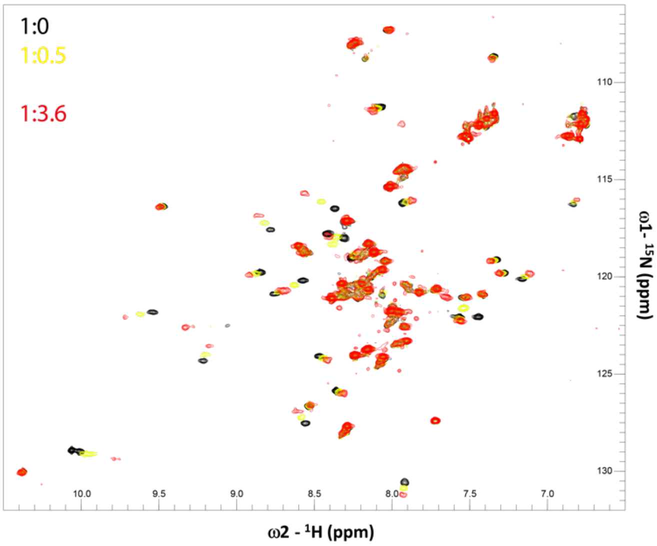

Supplementary Figure 24. NMR titrations of individual WW domains with Pro C region of SF1. ^{1}H-^{15}N HSQC spectra comparison of WW1 (141-175; up) and WW2 (176-236; down) constructs in free (black) and bound to GB1-SF1_ProC (yellow, orange and red) at different ratios. In both cases, fast exchange regimen is observed, indicating an affinity in the order of high micromolar to millimolar range of dissociation constants.

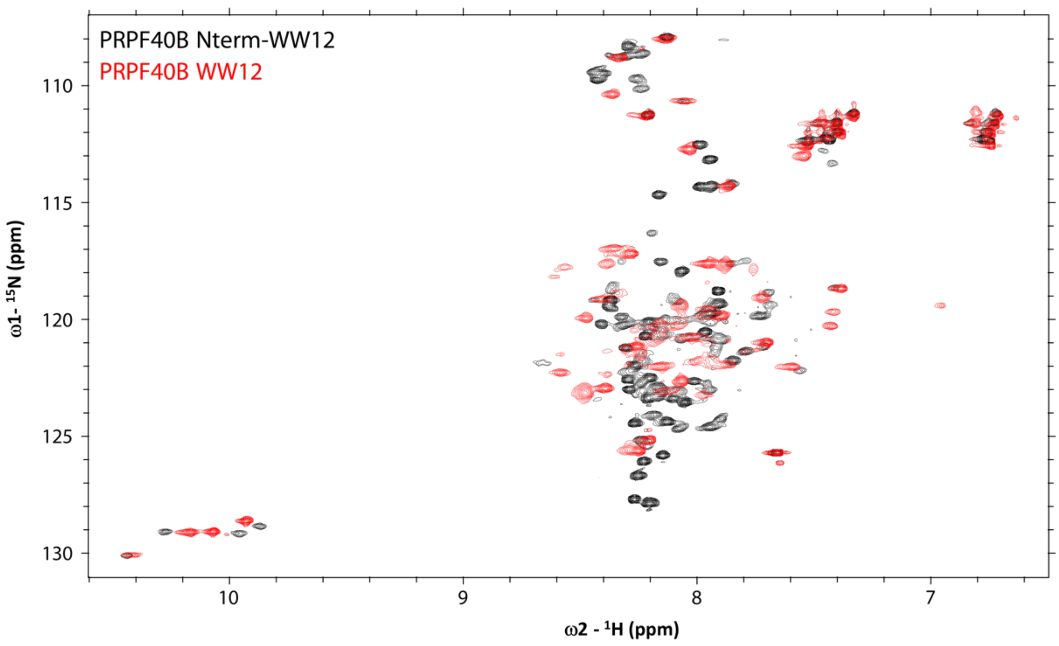

Supplementary Figure 25. PRPF40B N-terminal region affects also the spectra of the WW domains. ^{1}H-^{15}N HSQC spectra comparison of WW12 tandem construct (residues 90-181, in red) and N-terminal extended WW12 tandem (residues 1- 181, in black) of PRPF40B. Although the proteins do not show nice spectra (they miss many signals coming from the folded WW domains, probably due to exchange processes), the tryptophan side chains (4 Trp located within the WW domains) suffer mayor shifts when the N-terminal region is included. This presumably indicates the presence of similar intramolecular interactions (N-terminal proline rich region to WW domains) as in the homolog PRPF40A.

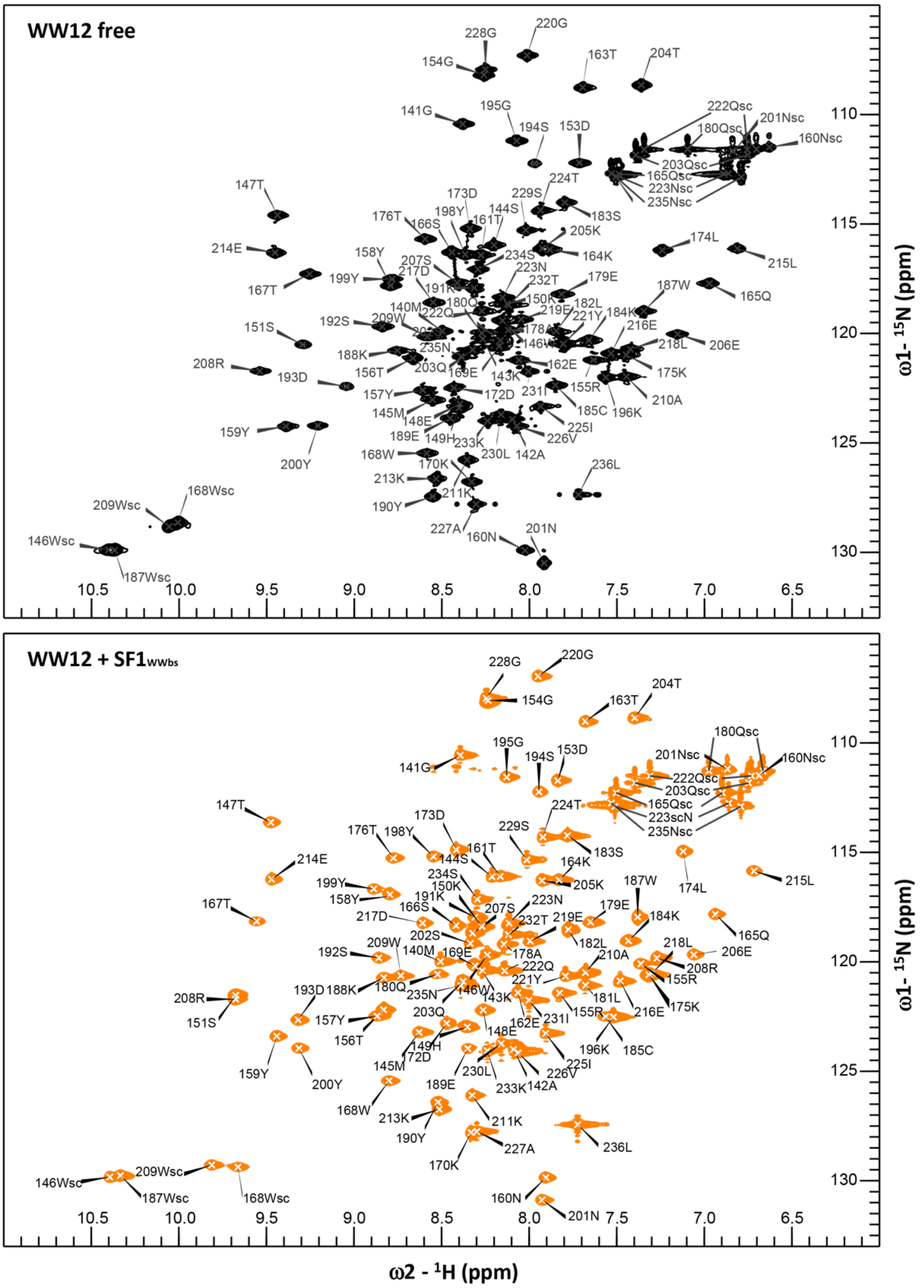

Supplementary Figure 26. Assigned ^{1}H-^{15}N HSQC spectra of PRPF40A WW12 construct free (up) and bound to SF1$_{WWbs}$ (1:2 molar ratio, down).

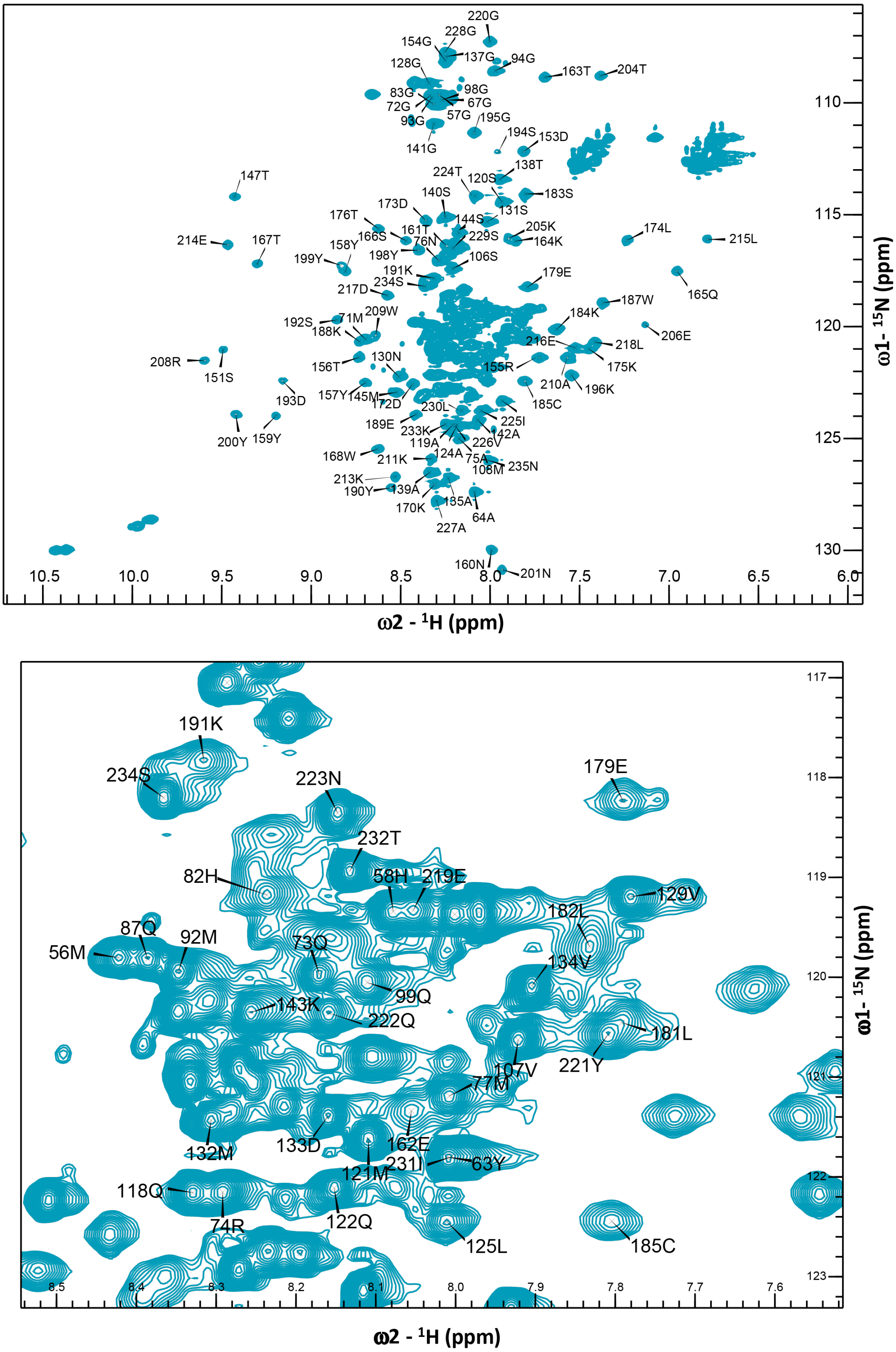

Supplementary Figure 27. Assigned ^{1}H-^{15}N HSQC spectrum of PRPF40A N-ext-WW12 construct. Showing in a zoom view the middle part of the spectrum.

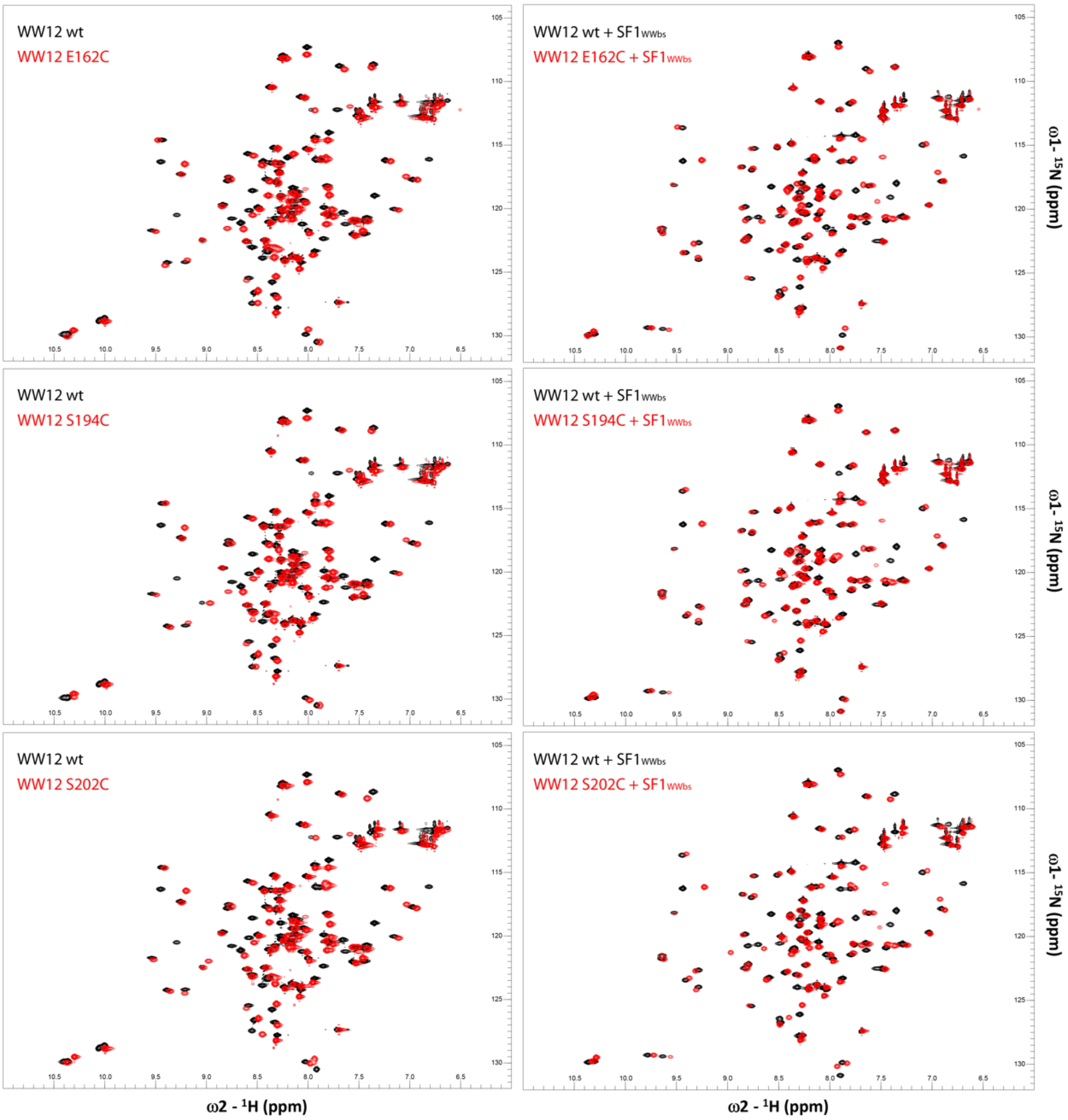

Supplementary Figure 28. The fold of WW12 mutants designed for PRE experiments is not affected, nor is their binding capacity. ^{1}H-^{15}N HSQC spectra comparison wild type WW12 tandem construct (black) with the corresponding mutants (red) in free (left) and in complex with $SF1_{WWbs}$ peptide (right).

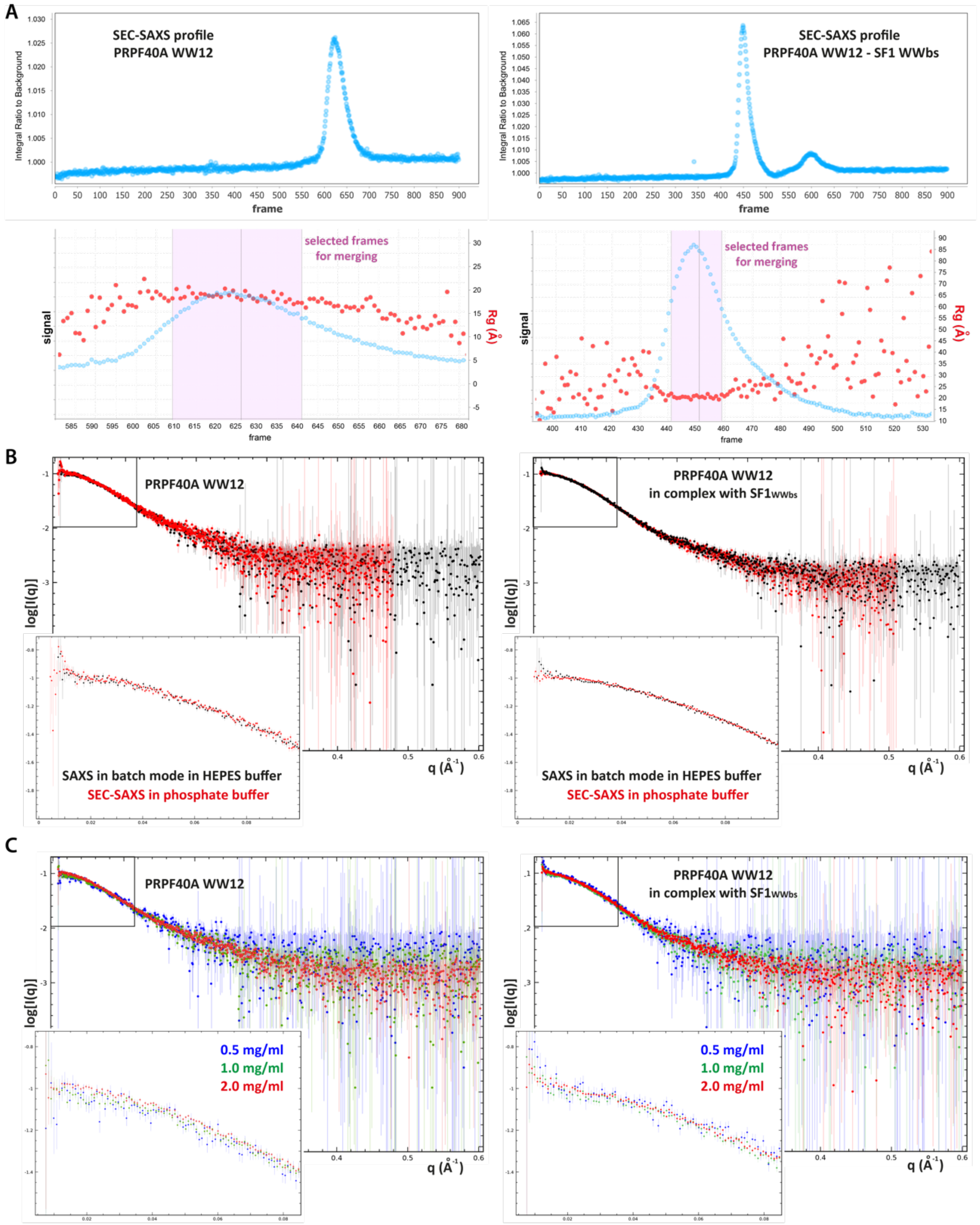

Supplementary Figure 29. SAXS data comparison. **A)** Complete SEC elution run of PRPF40A WW12 free and in complex with $SF1_{WWbs}$ (up) and calculated Rg values for each frame (down); the frames highlighted in purple were merged into the final SAXS curve. Notice the drift in the buffer signal in the SEC profiles, probably from capillary fouling due to the phosphate buffer. **B)** Comparison of the SAXS curves derived from SEC-SAXS in phosphate buffer and in batch mode acquired in HEPES buffer for both samples. Zoomed views in low angle values are included. **C)** Comparison of the SAXS profiles for PRPF40A WW12 free and in complex with $SF1_{WWbs}$ at 3 different concentrations (0.5 mg/ml – blue, 1 mg/ml – green and 2 mg/ml – red), including zoomed views for the low angle values. In all SAXS curves (**B & C**), dots indicate scattering intensity values and grey lines the measurement errors.

SUPPLEMENTARY TABLES

Supplementary Table 1. Statistics of structural calculations for the ensemble of 20 lowest-energy refined structures of free PRPF40A WW12.

**Experimental restraints**						
Distance restraints						
**Total NOE derived**	**2409**					
Unambiguous NOE	*1820*					
Intra-residue (|i-j|=0)	693					
Sequential (|i-j|=1)	391					
Medium-range (1<|i-j|<5)	255					
Long-range (|i-j|>4)	481					
Ambiguous NOE	*589*					
Dihedral angular restraints (TALOS)						
ϕ / ψ	71 / 71					
**Structural statistics**						
Violations						
Number of NOE violations > 0.2 Å	0.4 ± 0.4					
maximum NOE violation (Å)	0.31					
Number of dihedral violations > 5º	0.7 ± 0.7					
maximum dihedral violation (º)	8.3					
Deviations from idealized geometry						
Bond lengths (Å)	0.0050 ± 0.0001					
Bond angles (º)	0.578 ± 0.010					
Impropers (º)	1.42 ± 0.07					
Ramachandran Plot						
Most favorable regions	96.9%					
Additional allowed regions	3.1%					
Generously allowed regions	0.0%					
Disallowed regions	0.0%					
Average pairwise r.m.s.d (Å)						
WW1 (146-172)	bb	0.27 ± 0.07	//	heavy	1.03 ± 0.12	
WW2 (187-211)	bb	0.19 ± 0.05	//	heavy	0.90 ± 0.12	
WW12 (146-221)	bb	6.83 ± 3.68	//	heavy	7.08 ± 3.06	

Supplementary Table 2. SEC-SAXS derived structural parameters.

	**PRPF40A WW12**	**PRPF40A WW12-SF1$_{WWbs}$**
SAXS data collection		
Sample concentration	1.0 mg/ml	2.0 mg/ml
Temperature	25ºC	25ºC
Measured q-range	0.0075 – 0.6022 Å^{-1}	0.0075 – 0.6022 Å^{-1}
Guinier analysis (primus/qt – ATSAS 3.2.1)		
I(0)	0.097 ± 0.001 a.u.	0.110 ± 0.001 a.u.
Rg	20.1 ± 0.3 Å	19.4 ± 0.2 Å
s·Rg limits	0.18 - 1.29	0.21 – 1.29
Fidelity	0.62	0.49
PDDF/P(r) analysis (Scatter IV)		
I(0)	0.097 ± 0.010 a.u.	0.100 ± 0.005 a.u.
Rg	20.2 ± 0.7 Å	19.0 ± 0.3 Å
Dmax	63 Å	60 Å
q-range	0.008 – 0.310 Å^{-1}	0.010 – 0.545 Å^{-1}
P(r) fit assessment (Total quality estimate)	0.88	0.89
MW estimation (primus/qt – ATSAS 3.2.1)		
Theoretical (from sequence)	11.4 kDa	13.8 kDa
From Porod volume (MoW)	10.4 kDa	11.2 kDa
From volume of correlation (Vc)	11.9 kDa	12.0 kDa
**SASDBD ID**	**SASDxxx**	**SASDxxx**

Supplementary Table 3. Isothermal Titration Calorimetry Analysis Table

PRPF40A	GB1-peptide	Kd (μM)	N (sites)	ΔH (kJ/mol)
	Polyproline series			
WW12	P10	28.6 ± 5.6	0.89 ± 0.11	-40.3 ± 5.2
WW12	P13	8.54 ± 1.46	0.78 ± 0.07	-65.5 ± 6.45
WW12	P16	3.35 ± 0.27	0.88 ± 0.08	-63.6 ± 2.6
WW12	P19	6.76 ± 0.96	1.10 ± 0.04	-76.0 ± 3.5
WW12	P22	9.29 ± 0.88	1.39 ± 0.03	-77.9 ± 2.4
WW12	P25	10.8 ± 1.8	2.30 ± 0.09	-69.5 ± 2.7
WW12	P29	9.15 ± 1.85	2.47 ± 0.09	-65.9 ± 2.1
	SF1 16mers			
WW12	342 SAPRPAAPANNPPPPS 357	n.d.	n.d.	n.d.
WW12	387 PGGPGGGPHSFPHPLP 402	n.d.	n.d.	n.d.
WW12	470 MPPPPMGMMPPPPPPP 485	13.9 ± 2.3	0.90 ± 0.16	-57.1 ± 3.4
WW12	478 MPPPPPPPSGQPPPPP 493	9.59 ± 0.9	0.80 ± 0.11	-60.0 ± 4.0
WW12	425 MQPPPPPMNQGPHPPG 440	20.2 ± 3.1	0.87 ± 0.07	-55.8 ± 5.9
WW12	565 TMVPLPPGVQPPLPPG 580	30.4 ± 6.7	1.06 ± 0.11	-56.5 ± 8.2
WW12	596 MAYPPPPPPPPMDPSN 611	65.9 ± 50.7	1.10 ± 0.30	-45.8 ± 36.8
WW12	623 MPPFGMPPAPPPPPPQ 638	23.3 ± 13.3	0.95 ± 0.10	-44.8 ± 14.8
WW12	575 PPLPPGAPPPPPPPPP 590	1.40 ± 0.16	1.00 ± 0.03	-81.8 ± 1.7
N-ExtWW12	565 TMVPLPPGVQPPLPPG 580	n.d.	n.d.	n.d.
N-ExtWW12	470 MPPPPMGMMPPPPPPP 485	n.d.	n.d.	n.d.
N-ExtWW12	575 PPLPPGAPPPPPPPPP 590	8.71 ± 0.88	0.88 ± 0.03	-66.1 ± 3.7
Mut N-ExtWW12	575 PPLPPGAPPPPPPPPP 590	2.21 ± 0.24	0.84 ± 0.05	-87.8 ± 9.2
	SF1 longer constructs			
WW12	SF1 Pro A	25.1 ± 6.5	0.99 ± 0.17	-63.2 ± 14.8
WW12	SF1 Pro B	20.6 ± 4.9	1.15 ± 0.15	-72.5 ± 13.7
WW12	SF1 Pro C	2.02 ± 0.09	0.88 ± 0.01	-86.2 ± 0.9
WW12	SF1 Pro D	10.1 ± 4.2	0.87 ± 0.02	-66.9 ± 18.9
WW12	SF1 Pro ABCD	4.91 ± 0.51	0.28 ± 0.05	-280.5 ± 40.5
WW12	SF1 Pro BCD	3.65 ± 0.71	0.28 ± 0.02	-274.0 ± 36.0
WW12	SF1 Pro CD	3.80 ± 0.69	0.39 ± 0.02	-193.3 ± 13.0
WW12	SF1 Pro BC	6.83 ± 2.33	0.53 ± 0.05	-121.3 ± 6.9
	SF3A1 constructs			
WW12	SF3A1 Middle Pro	8.86 ± 1.38	0.56 ± 0.14	-114.0 ± 27.1
WW12	SF3A1 C-ter Pro	19.3 ± 4.5	0.27 ± 0.07	-288.0 ± 78.8

Supplementary Table 4. Statistics of structural calculations for the ensemble of 20 lowest-energy refined structures of the complex PRPF40A WW12 - $SF1_{WWbs}$.

**Experimental restraints**	**PRPF40A WW12**	**$SF1_{WWbs}$**
Distance restraints		
Total NOE derived	3224	
Unambiguous NOE	2849	154
Intra-residue (|i-j|=0)	915	99
Sequential (|i-j|=1)	556	55
Medium-range (1<|i-j|<5)	461	0
Long-range (|i-j|>4)	917	0
Intermolecular NOE	261	
Ambiguous NOE	221	
Dihedral angular restraints (TALOS)		
ϕ / ψ	71 / 71	13/13
Residual Dipolar Couplings (RDCs)		
$^{1}D(N,H)$, Pf1	61	0
**Structural statistics**		
Violations		
Number of NOE violations > 0.2 Å	0.5 ± 0.5	
maximum NOE violation (Å)	0.36	
Number of dihedral violations > 5º	1.9 ± 0.8	
maximum dihedral violation (º)	8.0	
Cornilescu Q factor for RDCs	0.293 ± 0.005	
Deviations from idealized geometry		
Bond lengths (Å)	0.0070 ± 0.0001	
Bond angles (º)	0.727 ± 0.010	
Impropers (º)	1.78 ± 0.06	
Ramachandran Plot		
Most favorable regions	92,8%	
Additional allowed regions	7.2%	
Generously allowed regions	0.0%	
Disallowed regions	0.0%	
Average pairwise r.m.s.d (Å)		
WW1 (146-172) + SF1 (575-582)	bb 0.25 ± 0.07 // heavy 0.85 ± 0.14	
WW2 (187-211) + SF1 (586-590)	bb 0.25 ± 0.07 // heavy 0.74 ± 0.11	
WW12 (146-221) + SF1 (575-582,586-590)	bb 0.77 ± 0.23 // heavy 1.16 ± 0.20	

Supplementary Table 5. All proteins enriched in PRPF40A-Nter-MUT immuno-precipitation compared to PRPF40A-Nter_WT; corresponding to blue in **Figure 5D**. Proteins significantly enriched in either PRPF40A-Nter-WT or either PRPF40A-Nter-MUT (Supplementary Data) were compared and ranked according to the enrichment values (log2(FC)) and its probability (-log10(p-value)). Adjusted p-values (two-sided) were determined by linear modeling using the limma package. We determined Pro-rich regions by the presence of at least two consecutive clusters of proline residues (3 proline residues in 5 positions) in tracts of 40 residues long.

Gene names	log2(FC)	-log10 (p-value)	Pro-rich region?	High affinity motif sequence	Unique peptides found
WIZ	1,529	2,216	Yes	-	6
LARP7	1,221	2,140	No	-	10
NKRF	1,452	2,848	No	-	6
CCNT1;CCNT2	1,335	2,476	Yes	PPLP, PPPP	17
DIAPH2	1,317	2,422	Yes	PPLP, PPPP	16
SNRNP200	1,210	2,106	Yes	-	11
MTREX	1,198	2,072	No	-	17
CDK9	1,401	2,683	No	-	7
SF3A3	1,179	2,021	No	-	6
SF3A2	1,275	2,295	Yes	PPLP, PPPP	7
SF3A1	1,381	2,618	Yes	PPLP, PPPP	14
MEPCE	1,321	2,432	Yes	PPPP	5
CDK12	1,215	2,121	Yes	PPLP, PPPP	8
ATXN10	1,570	2,316	No	-	3
HNRNPUL1	1,072	1,735	Yes	PPPP	9
SAE1	1,104	1,324	No	-	4
INF2	1,013	1,586	Yes	PPLP, PPPP	23
DDX20	1,113	1,841	No	-	3
SF3B1	0,993	1,538	No	-	30
ACTR2	0,913	1,350	No	-	7
SF3B2	0,897	1,313	Yes	PPPP	15
EFTUD2	1,181	1,469	No	-	5
SF3B3	0,897	1,314	No	-	14
SF1	0,962	1,465	Yes	PPLPPGAP, PPPP	2/6
RBM17	1,293	1,694	No	-	3
ATAD3A	1,016	1,594	Yes	PPLP	4
FAM120A	1,247	1,600	Yes	PPLP, PPPP	1

Supplementary Table 6. List of DNA oligonucleotides used in this work.

Primer name	Sequence (5´→ 3´)
FBP11_1_Fw	AGCACCATGGAAATGCGCCCAGGGACG
FBP11_310_Rv	AGCATGGTACCTTACACAGTGCCGGAC
FBP11_141_Fw	GGCGCCATGGGTGCAAAATCAATGTGGAC
FBP11_223_Rv	TCCGGTACCTTAGTTCTGATAACCTTCCAG
FBP11_236_Rv	GCTCGGTACCTTACAGGTTAGATTTCG
FBP11_248_Rv	GATCCGGTACCTTACTGTTTACTAGACTC
FBP11_56_Fw	TTCAGGGCGCCATGGGACATCCCGGTATGC
SF1_410_Fw	GGCGCCATGGGTCATCCCATGCAGC
SF1_468_Rv	TCCGGTACCTTAACCCATAGGCGGTGG
SF1_468_Fw	GGCGCCATGGGTATGATGCCTCC
SF1_539_Rv	TCCGGTACCTTAGCCTGCACTCGTCGTCG
SF1_539_Fw	GGCGCCATGGGCACCGGGTCTATCCC
SF1_594_Rv	TCCGGTACCTTATCCAGCGCTTCCTGG
SF1_594_Fw	GGCGCCATGGGAATGATGTACGC
SF1_639_Rv	TCCGGTACCTTAATTTTGGGGAGG
SF3A1_364_Fw	AGCACCATGGAGGGGCAAAAGG
SF3A1_413_Rv	AGCATGGTACCTTAAGAAACCAAATATTCATCTGG
SF3A1_552_Fw	AGCACCATGGAAATTCCACAACAGCC
SF3A1_683_Rv	AGCATGGTACCTTATTTTGAGGTAGGCTCGTCC
P6_Fw	CATGTCTGGAAGTCCTCCGCCACCACCTCCATCTGGAAGTGGCAATTAAGTAC
P6_Rv	TTAATTGCCACTTCCAGATGGAGGTGGTGGCGGAGGACTTCCAGA
P8_Fw	CATGTCTGGAAGTCCTCCGCCACCACCTCCGCCTCCATCTGGAAGTGGCAATTAAGTAC
P8_Rv	TTAATTGCCACTTCCAGATGGAGGCGGAGGTGGTGGCGGAGGACTTCCAGA
PP_end_Fw (10,13,16)	CCACCTCCGCCTCCATCTGGAAGTGGCAATTAAGTAC
PP_end_Rv (10,13,16)	TTAATTGCCACTTCCAGATGGAGGCGGA
P10_Fw	CATGTCTGGAAGTCCTCCGCCACCACCT
P10_Rv	GGTGGAGGTGGTGGCGGAGGACTTCCAGA
P13_Fw	CATGTCTGGAAGTCCTCCGCCACCGCCACCACCTCCT
P13_Rv	GGTGGAGGAGGTGGTGGCGGTGGCGGAGGACTTCCAGA
P16_Fw	CATGTCTGGAAGTCCTCCGCCACCACCACCTCCGCCGCCTCCTCCT
P16_Rv	GGTGGAGGAGGAGGCGGCGGAGGTGGTGGTGGCGGAGGACTTCCAGA
MiddlePro_Fw (19-29)	CCACCTCCACCTCCGCCTCCACCACCG
MiddlePro_Rv (19-29)	TGGAGGCGGTGGTGGAGGCGGAGGTGGA
SF1_16merA_Fw1	CATGTCTGGAAGTTCTGCGCCACGTCCAGCAGCACC
SF1_16merA_Fw2	TGCGAACAATCCACCACCGCCGTCTTCTGGAAGTGGCAATTAAGTAC
SF1_16merA_Rv1	GTTCGCAGGTGCTGCTGGACGTGGCGCAGAACTTCCAGA
SF1_16merA_Rv2	TTAATTGCCACTTCCAGAAGACGGCGGTGGTGGATT
SF1_16merB_Fw1	CATGTCTGGAAGTCCGGGTGGACCAGGCGGAGGGCCTC
SF1_16merB_Fw2	ATAGCTTCCCGCACCCGCTGCCTTCTGGAAGTGGCAATTAAGTAC
SF1_16merB_Rv1	CTCCGCCTGGTCCACCCGGACTTCCAGA
SF1_16merB_Rv2	TTAATTGCCACTTCCAGAAGGCAGCGGGTGCGGGAAGCTATGAGGCC
SF1_16merD_Fw1	CATGTCTGGAAGTATGCAGCCTCCTCCCCCACCGATGAATCAGGGGCCACAT
SF1_16merDG_Fw2	CCTCCGGGCTCTGGAAGTGGCAATTAAGTAC
SF1_16merD_Rv1	CGGAGGATGTGGCCCCTGATTCATCGGTGGGGGAGGAGGCTGCATACTTCCAGA
SF1_16merDG_Rv2	TTAATTGCCACTTCCAGAGCC
SF1_16merG_Fw1	CATGTCTGGAAGTACCATGGTGCCTTTGCCTCCGGGAGTCCAACCACCACTG
SF1_16merG_Rv1	CGGAGGCAGTGGTGGTTGGACTCCCGGAGGCAAAGGCACCATGGTACTTCCAGA
SF1_16merE_Fw1	CATGTCTGGAAGTATGCCCCCGCCGCCAATGGGGATGATGCCTCCG
SF1_16merEFH_Fw2	CCTCCACCTCCACCGTCTGGAAGTGGCAATTAAGTAC
SF1_16merE_Rv1	TGGAGGCGGAGGCATCATCCCCATTGGCGGCGGGGGCATACTTCCAGA
SF1_16merEFH_Rv2	TTAATTGCCACTTCCAGACGGTGGAGG
SF1_16merF_Fw1	CATGTCTGGAAGTATGCCGCCGCCTCCGCCGCCCCCGTCCGGCCA
SF1_16merF_Rv1	TGGAGGTTGGCCGGACGGGGGCGGCGGAGGCGGCGGCATACTTCCAGA
SF1_16merH_Fw1	CATGTCTGGAAGTCCTCCCCTTCCTCCGGGTGCACCACCGCCACCT
SF1_16merH_Rv1	TGGAGGAGGTGGCGGTGGTGCACCCGGAGGAAGGGGAGGACTTCCAGA
SF1_16merI_Fw1	CATGTCTGGAAGTATGTATGCGCCACCGCCACCACCTCCG
SF1_16merI_Fw2	CCTCCTATGGACCCTAGTAATTCTGGAAGTGGCAATTAAGTAC
SF1_16merI_Rv1	TGGTGGCGGTGGCGCATACATACTTCCAGA
SF1_16merI_Rv2	TTAATTGCCACTTCCAGAATTACTAGGGTCCATAGGAGGCGGAGG
SF1_16merJ_Fw1	CATGTCTGGAAGTATGCCTCCGTTCGGCATGCCGCCTGCCCCTCC
SF1_16merJ_Fw2	TCCACCTCCTCCGCAATCTGGAAGTGGCAATTAAGTAC
SF1_16merJ_Rv1	GGCAGGCGGCATGCCGAACGGAGGCATACTTCCAGA
SF1_16merJ_Rv2	TTAATTGCCACTTCCAGATTGCGGAGGAGGTGGAGGAGG
Hela_amplification_1_FW	GTGGAGGCCTCATGATGGG
Hela_amplification_1_RV	CCAGCGAGATTCTTTTGTTTGAG

Hela_amplification_2_FW	ACAAAAGAATCTCGCTGGGC
Hela_amplification_2_RV	CCACACTTTGATCCTGAATAGCA
pAB013_lin_backbone_FW	TAATAGGGGCCCGTTTAAAC
pAB013_lin_backbone_RV	GGAACCTCCAGATCCACC
PRPF40A_lin_insert_FW	CAGGTGGATCTGGAGGTTCCATGTGTAGCGGCAGTGGC
PRPF40A_lin_insert_RV	GTTTAAACGGGCCCCTATTATTGCTTACTGCTTTCTTCAGCTTTG
Mutagenesis_1_FW	GCAGCTCATGGAATGATGCCGCAG
Mutagenesis_1_RV	AGCAGCCATATTCGCTCTCTGACC
Mutagenesis_2_FW	AGCGGCAGGAGTAAATAGTATGGATGTAGC
Mutagenesis_2_RV	GCGGCAGCCTGCATGGAAGCCTGAGA
