# Supplementary material for: Intramolecular autoinhibition regulates the selectivity of PRPF40A tandem WW domains for proline-rich motifs
Source: Nat Commun. 2024 May 8;15:3888. doi: 10.1038/s41467-024-48004-x (PMC11079029; doi:10.1038/s41467-024-48004-x)
Supplement: Supplementary file 3 — Reporting Summary [file 41467_2024_48004_MOESM3_ESM.pdf]

nature portfolio

Corresponding author(s): Santiago Martínez Lumbreras
Michael Sattler

Last updated by author(s): 12.04.2024

Reporting Summary

Nature Portfolio wishes to improve the reproducibility of the work that we publish. This form provides structure for consistency and transparency in reporting. For further information on Nature Portfolio policies, see our Editorial Policies and the Editorial Policy Checklist.

Statistics

For all statistical analyses, confirm that the following items are present in the figure legend, table legend, main text, or Methods section.

n/a	Confirmed	
☐	☒	The exact sample size (*n*) for each experimental group/condition, given as a discrete number and unit of measurement
☐	☒	A statement on whether measurements were taken from distinct samples or whether the same sample was measured repeatedly
☐	☒	The statistical test(s) used AND whether they are one- or two-sided<br>*Only common tests should be described solely by name; describe more complex techniques in the Methods section.*
☒	☐	A description of all covariates tested
☐	☒	A description of any assumptions or corrections, such as tests of normality and adjustment for multiple comparisons
☐	☒	A full description of the statistical parameters including central tendency (e.g. means) or other basic estimates (e.g. regression coefficient) AND variation (e.g. standard deviation) or associated estimates of uncertainty (e.g. confidence intervals)
☐	☒	For null hypothesis testing, the test statistic (e.g. F, t, r) with confidence intervals, effect sizes, degrees of freedom and P value noted<br>*Give P values as exact values whenever suitable.*
☒	☐	For Bayesian analysis, information on the choice of priors and Markov chain Monte Carlo settings
☒	☐	For hierarchical and complex designs, identification of the appropriate level for tests and full reporting of outcomes
☒	☐	Estimates of effect sizes (e.g. Cohen's d, Pearson's r), indicating how they were calculated

Our web collection on statistics for biologists contains articles on many of the points above.

Software and code

Policy information about availability of computer code

Data collection	TopSpin 4.1.0 (NMR acquitision, Bruker)<br>BsxCuBE (SAXS acquisition - ESRF)<br>SAXSLab 3.02 (SAXS acquisition - Rigaku)<br>Malvern PEAQ-ITC Analysis 1.21. (ITC acquisition)
Data analysis	NMRpipe 8.9.(NMR processing)<br>CCPNanalysis 2.5. (NMR analysis)<br>ARIA2.3 (Structure calculation)<br>ATSAS suite 3.1.3 (SAXS analysis)<br>ScAtter IVb (SAXS analysis)<br>Malvern PEAQ-ITC Analysis 1.21 (ITC analysis)<br>MaxQuant software package 2.1.3.0 (Mass spetrometry processing and analysis)<br>RStudio 2023.03.0 (Mass spectrometry analysis)<br>Pymol 2.3.1<br>Molmol 2K.2

For manuscripts utilizing custom algorithms or software that are central to the research but not yet described in published literature, software must be made available to editors and reviewers. We strongly encourage code deposition in a community repository (e.g. GitHub). See the Nature Portfolio guidelines for submitting code & software for further information.

Data

Policy information about availability of data

All manuscripts must include a data availability statement. This statement should provide the following information, where applicable:

- Accession codes, unique identifiers, or web links for publicly available datasets
- A description of any restrictions on data availability
- For clinical datasets or third party data, please ensure that the statement adheres to our policy

NMR chemical shifts for PRPF40A tandem of WW domains free and in complex with the SF1WWbs peptide, as well as the backbone chemical shifts of N-terminal extended WW12 construct, are deposited in the BMRB under accession codes 34839 [https://dx.doi.org/10.13018/BMR34839] – WW12 apo, 34840 [https://dx.doi.org/10.13018/BMR34840] – WW12/SF1 complex, and 52046 [https://dx.doi.org/10.13018/BMR52046] – N-ext-WW12. The structure ensembles of WW12 free and bound to SF1WWbs are deposited in the PDB with accession codes 8PXW [https://doi.org/10.2210/pdb8PXW/pdb] and 8PXX [https://doi.org/10.2210/pdb8PXX/pdb], respectively. SAXS data are deposited in the SASBDB with accession codes: SASDSK7 [https://www.sasbdb.org/data/SASDSK7/] and SASDSL7 [https://www.sasbdb.org/data/SASDSL7/] for the SEC-SAXS derived data of WW12 free and in complex with SF1WWbs, respectively; SASDUH4 [https://www.sasbdb.org/data/SASDUH4/] and SASDUJ4 [https://www.sasbdb.org/data/SASDUJ4/] for the batch mode derived curves of free and bound PRPF40A WW12. The mass spectrometry proteomics data have been deposited to the ProteomeXchange Consortium via PRIDE partner repository with the dataset identifier PXD046164 [http://www.ebi.ac.uk/pride/archive/projects/PXD046164]. The coordinates of the truncated PRPF40A WW tandem and the WW tandem of the yeast homolog Prp40 are publicly available in the PDB under the codes 2L5F [https://doi.org/10.2210/pdb2L5F/pdb] and 1O6W [https://doi.org/10.2210/pdb1O6W/pdb] respectively. Source data are provided as a Source Data file.

Research involving human participants, their data, or biological material

Policy information about studies with human participants or human data. See also policy information about sex, gender (identity/presentation), and sexual orientation and race, ethnicity and racism.

Reporting on sex and gender	N/A
Reporting on race, ethnicity, or other socially relevant groupings	N/A
Population characteristics	N/A
Recruitment	N/A
Ethics oversight	N/A

Note that full information on the approval of the study protocol must also be provided in the manuscript.

Field-specific reporting

Please select the one below that is the best fit for your research. If you are not sure, read the appropriate sections before making your selection.

[x] Life sciences [ ] Behavioural & social sciences [ ] Ecological, evolutionary & environmental sciences

For a reference copy of the document with all sections, see nature.com/documents/nr-reporting-summary-flat.pdf

Life sciences study design

All studies must disclose on these points even when the disclosure is negative.

Sample size	For ITC data, at least two replicates were performed. Three biological replicates were done for Immunoprecipitation experiments.
Data exclusions	No data were excluded.
Replication	3 biological sample replicates were used for Immunoprecipitation with high degree of convergence. We selected the proteins identified in at least two out of the three replicates. The ITC data was typically highly reproducible between replicates.
Randomization	There was no allocation of groups done in this study and therefore no randomization was required
Blinding	There was no allocation of groups done in this study and therefore no blinding was required

Reporting for specific materials, systems and methods

We require information from authors about some types of materials, experimental systems and methods used in many studies. Here, indicate whether each material, system or method listed is relevant to your study. If you are not sure if a list item applies to your research, read the appropriate section before selecting a response.

Materials & experimental systems

n/a	Involved in the study
☐	☒ Antibodies
☐	☒ Eukaryotic cell lines
☒	☐ Palaeontology and archaeology
☒	☐ Animals and other organisms
☒	☐ Clinical data
☒	☐ Dual use research of concern
☒	☐ Plants

Methods

n/a	Involved in the study
☒	☐ ChIP-seq
☒	☐ Flow cytometry
☒	☐ MRI-based neuroimaging

Antibodies

Antibodies used	Alpaca-anti-GFP nanobody conjugated to agarose beads. RRID: AB_2631357. Supplier: Chromotek. Catalog No: gta-20.
Validation	Validation and References can be found on the suppliers website https://www.ptglab.com/products/GFP-Trap-Agarose-gta.htm.

Eukaryotic cell lines

Policy information about cell lines and Sex and Gender in Research

Cell line source(s)	HeLa ATCC Cat# CCL-2, RRID:CVCL_0030
Authentication	Cell lines are commercial and not authenticated by us. ATCC comprehensively performs authentication and quality-control on all distribution lots of cell lines.
Mycoplasma contamination	The cell line was tested negative for mycoplasma before first usage.
Commonly misidentified lines (See ICLAC register)	No commonly misidentified cell lines were used in the study.

Plants

Seed stocks	N/A
Novel plant genotypes	N/A
Authentication	N/A
